# Generation of Binary Tree-Child phylogenetic networks

**DOI:** 10.1371/journal.pcbi.1007347

**Published:** 2019-09-11

**Authors:** Gabriel Cardona, Joan Carles Pons, Celine Scornavacca

**Affiliations:** 1 Department of Mathematics and Computer Science, University of the Balearic Islands, Ctra. de Valldemossa Ctra. de Valldemossa km. 7.5. 07122 - Palma, Spain; 2 Institut des Sciences de l’Evolution (ISE-M), Université de Montpellier, CNRS, IRD, EPHE, 34095 Montpellier Cedex 5, France; University of Basel, SWITZERLAND

## Abstract

Phylogenetic networks generalize phylogenetic trees by allowing the modelization of events of reticulate evolution. Among the different kinds of phylogenetic networks that have been proposed in the literature, the subclass of binary tree-child networks is one of the most studied ones. However, very little is known about the combinatorial structure of these networks. In this paper we address the problem of generating all possible binary tree-child (BTC) networks with a given number of leaves in an efficient way via reduction/augmentation operations that extend and generalize analogous operations for phylogenetic trees, and are biologically relevant. Since our solution is recursive, this also provides us with a recurrence relation giving an upper bound on the number of such networks. We also show how the operations introduced in this paper can be employed to extend the evolutive history of a set of sequences, represented by a BTC network, to include a new sequence. An implementation in python of the algorithms described in this paper, along with some computational experiments, can be downloaded from https://github.com/bielcardona/TCGenerators.

## Author summary

Phylogenetic networks are widely used to represent evolutionary scenarios with reticulated events, and among them, the class of binary tree-child (BTC for short) networks is one of the most studied ones. Despite its importance, BTC networks, as mathematical objects, are not yet fully understood. In this paper we introduce two operations (reduction and augmentation) on the set of BTC networks that generalize well known operations on phylogenetic trees, and show how they can be used to analyze and synthesize any BTC network. Apart from the mathematical formulation of the problem, we exhibit how these operations can be used in biological applications to add a new sequence to a given BTC network. This can be useful, for instance, to update the network without redoing the whole search, or in a phylogenetic placement perspective. We also obtain a recursive formula for a bound on the number of such networks. We have implemented the algorithms in this paper, made them available on a public repository, and used this implementation to perform some computational simulations.

## Introduction

Phylogenetic networks are, mathematically, a generalization of phylogenetic trees that, containing nodes with more than one ancestor, permit to model reticulated evolutionary events such as recombinations, lateral gene transfers and hybridizations. We note here that other representations, for example gene tree-species tree reconciliations [1], permit to model scenarios including other classes of evolutionary events such as duplications, losses and transfers of genes.

In this paper, we shall focus on directed phylogenetic networks (see [2] for a short survey on the phylogenetic network paradigm also covering undirected phylogenetic networks). Mathematically, such networks are, in the broadest sense, directed acyclic graphs with a single node with no incoming arcs –the *root*– representing the common ancestor of all the Operational Taxonomic Units (OTUs for short) under study, which are represented by the nodes with no outgoing arcs –the *leaves*– of the graph; internal nodes represent either (hypothetical) speciations or (hypothetical) reticulated events. Nodes with a single incoming arc –*tree* nodes– model extant or non-extant OTUs, and arcs between tree nodes model direct descent through mutation; nodes with two incoming arcs –*hybrid* nodes– model reticulated events involving the OTUs corresponding to the two parents of the node under consideration, and whose resulting OTU is modeled as its single child. Unfortunately, this definition is too broad, both for representing biologically-meaningful evolutionary scenarios, and for giving objects that can be efficiently handled.

So far, several restrictions on this general definition have been introduced in the literature. A few of them are based on biological considerations, while the majority have been introduced to artificially narrow the space of networks under study. This led to the introduction of a panoply of different classes of phylogenetic networks, such as time-consistent networks [3], regular networks [4], orchard networks [5], galled trees [6] and galled networks [7], level-*k* networks [8], tree-sibling networks [9], tree-based networks [10] and LGT networks [11], just to name a few.

**Fig 1:**
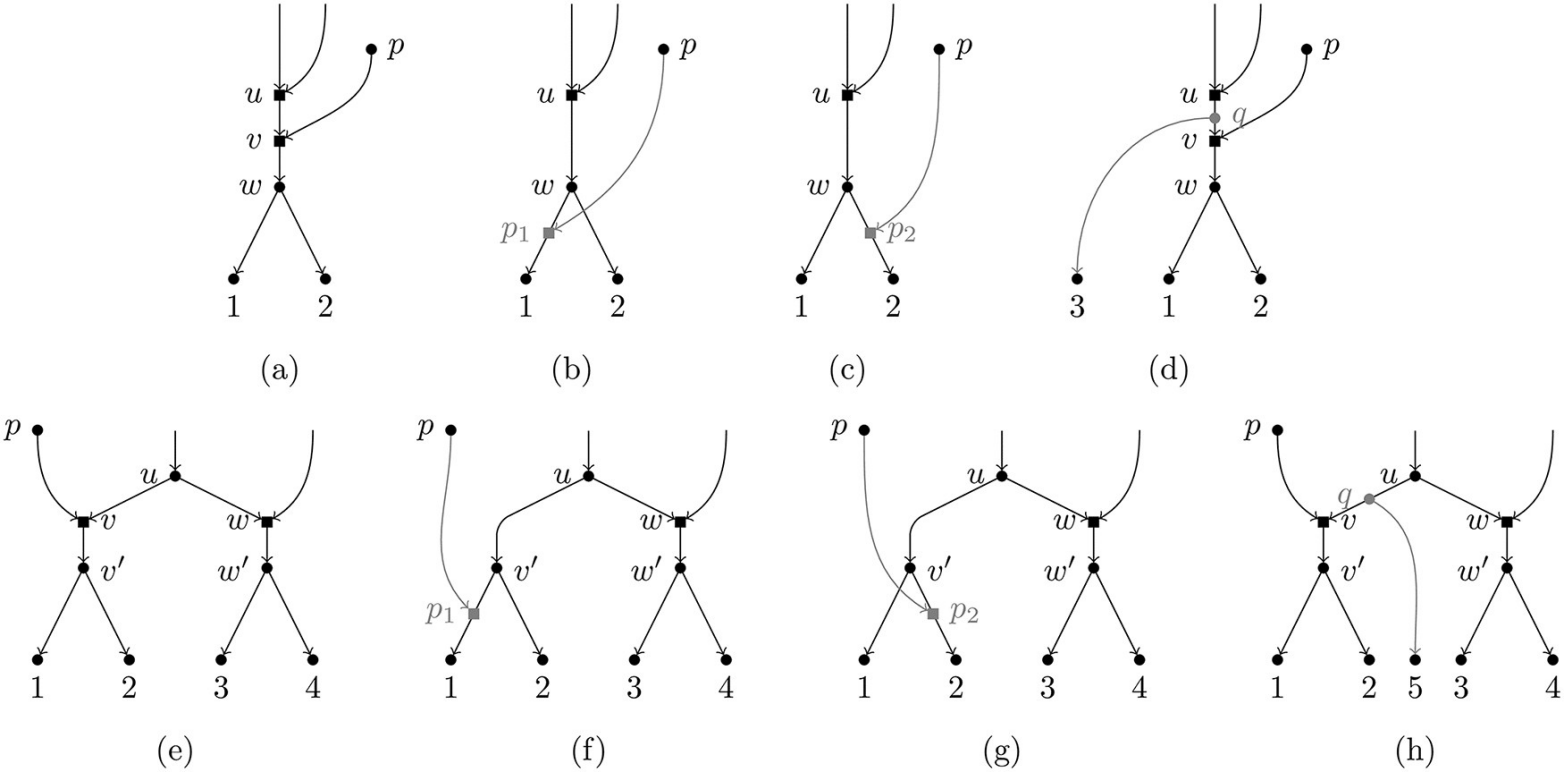
Limitations of BTC networks. The scenarios in (a) and (e) are not BTC networks since in both cases the node labeled with *u* has no tree-node child. Still, the scenario in (a) can be approximated either by the scenario in (b) or by that in (c), both scenarios being BTC networks. Also, if we are lucky enough to find an OTU between the hybrid event represented by the node *u* and that represented by *v*, e.g. the node *q* in (d), then the hybrid event in *v* can be modeled. The same reasoning holds for the scenario in (e) and those in (f,g,h). Thus, if the “true” network is not BTC, we can always find a BTC network those topology is not far from the true one. In our example, the networks in (b,c) are both a head-moving rSPR [16] away from the true network in (a). The same holds for the networks in (f,g) w.r.t. the one in (e). In general, each violation of the TC property, i.e. each hybrid node that has only hybrid children, moves the reconstructible network a head-moving rSPR away from the true one. Note that the configuration in (a) is known to generate severe indistinguishability issues [17].

In this paper, we shall focus on binary tree-child networks (BTC networks, for short), which were introduced by [9] and are one of the most studied classes of phylogenetic networks [12–15]. Mathematically, being tree-child means that every internal node is compelled to have at least a child node that is a tree node. BTC networks have been introduced in order to adjust a complex biological reality in a computationally tractable way. Although the original motivation for these networks is not biological, and hence they present some limitations, the mathematical constraint on BTC networks translates biologically as follows: every non-extant OTU is required to have at least an offspring species that evolved only through mutation. This means that not all biologically-meaningful evolutionary scenarios can be modeled with BTC networks. For example, the scenarios depicted in Fig 1(a) and 1(e) are not allowed since, in these cases, the node labeled with *u* has no child with a single incoming arc. Still, BTC networks are one of the most permissive classes of phylogenetic networks and they permit to model quite a lot of meaningful scenarios, and those that cannot be modeled can be approximated pretty well, see Fig 1.

The combinatorial study of phylogenetic networks is nowadays a challenging and active field of research. Nevertheless, the problem of counting how many phylogenetic networks are in a given subclass of networks is still open even for long-established classes. More precisely, this problem has been only recently solved for galled networks [18]; for other classes, including tree-child networks, we only have asymptotic results [19, 20]. Associated to the problem of counting networks, we find the problem of their “injective” generation, i.e. without having to check for isomorphism between pairs of constructed networks.

The main result of this paper is a systematic way of recursively generating, with unicity, all BTC networks with a given number of leaves. This generation relies on a pair of reduction/augmentation operations –both producing BTC networks– where reductions decrease by one the number of leaves in a network, and augmentations increase it. The idea of using pairs of operations has already been used to deal either with other classes of phylogenetic networks [21, 22], or for BTC networks but without the unicity feature [5].

In order to give a biological meaning to these augmentation operations, assume that the evolutive history of a given group of species is known and modeled by a BTC network, and a new species has to be taken into account. The augmentation operation determines exactly how the phylogenetic network has to be modified, and what is the minimum information needed to establish this modification, in order to model the evolution of the group of species with the newly incorporated one.

As an interesting side product, this procedure gives a recursive formula providing an upper bound on the number of BTC networks. Note also that being able to generate all BTC networks with a given number of leaves may also be interesting as part of a divide-and-conquer framework to reconstruct phylogenetic networks, where we start by computing BTC networks on 3/5 leaves that are then combined together, as done for example in [23, 24].

The paper is organized as follows. In Section Methods, we review the basic definitions that will be used throughout the paper. The main part of the paper is in Section Results, which is split between different subsections. Subsection Reduction of networks is devoted to the reduction procedure, while in Subsection Generation of networks we introduce the augmentation operation and prove that any BTC network can be obtained, in a unique way, via a sequence of augmentation operations applied to the trivial network with one leaf. In Subsection Bounding the number of networks, we show how to relax the conditions for the applicability of the augmentation operation to obtain a recursive formula providing an upper bound on the number of BTC networks. In Subsection An application to phylogenetic reconstruction, we give a concrete biological application of the methods we have developed. In Subsection Computational experiments, we introduce the implementation of the algorithms presented in the paper, and some experimental results, including the exhaustive generation of all BTC networks with up to six leaves and an upper bound of their number up to ten leaves. Finally, in Section Discussion we discuss how our reduction/augmentation operations extend and generalize analogous operations for phylogenetic trees.

## Methods

In this section we introduce the mathematical notations that are used in the rest of the paper.

Throughout this paper, a *tree node* in a directed graph is a node *u* whose pair of degrees *d*(*u*) = (indegree *u*, outdegree *u*) is (1, 0) for the *leaves*, (0, 2) for the *roots*, or (1, 2) for *internal* tree nodes; a *hybrid node* is a node *u* with *d*(*u*) = (2, 1). If two nodes *u* and *v* are linked by an arc (*u*, *v*) we say that *u* is a *parent* of *v*, or that *v* is a *child* of *u*. Also, two nodes are *siblings* if they have a common parent.

A *binary phylogenetic network* over a set *X* of taxa is a directed acyclic graph with a single root such that all its nodes are either tree nodes or hybrid nodes, and whose leaf set is bijectively labeled by the set *X*. In the following, we will implicitly identify every leaf with its label. A binary phylogenetic network is *tree-child* if every node either is a leaf or has at least one child that is a tree node [9]; in particular, the single child of a hybrid node must be a tree node. We will denote by ${BTC}_{n}$ the set of binary tree-child phylogenetic networks over the set [*n*] = {1, …, *n*}.

An *elementary node* in a directed graph is a node *u* with *d*(*u*) = (1, 1) or *d*(*u*) = (0, 1). An *elementary path* *p* is a path *u*_1_, …, *u*_*k*_ composed of elementary nodes such that neither the single parent of *u*_1_ (if it exists) nor the single child of *u*_*k*_ are elementary. We call these last two nodes respectively the *grantor* (if this node is well-defined) and *heir* of the nodes in the elementary path. In case of an elementary node, its grantor and heir are those of the nodes in the single elementary path that contains the given node. The *elimination* of an elementary path *p* consists in deleting all nodes in *p*, together with their incident arcs, and adding an arc between the grantor and the heir of *p* (provided that the grantor exists; otherwise, no arc is added). The elimination of an elementary node is defined as the elimination of the elementary path that contains the given node.

**Fig 2:**
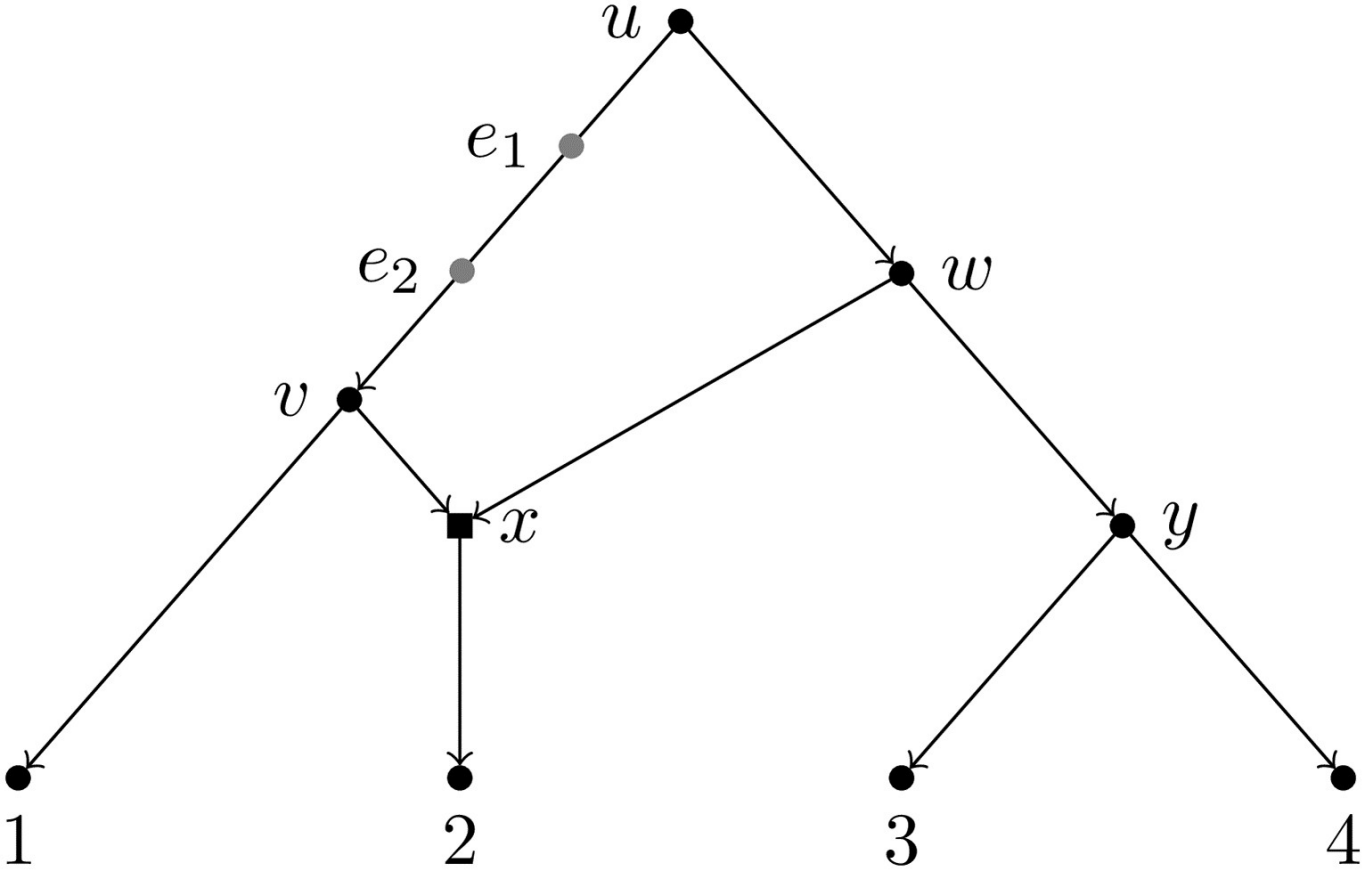
The definitions introduced in the methods section. For the network *N* in the figure (black nodes and arcs only), we have the following: *X* = {1, 2, 3, 4} is the set of taxa, *u* is the root, *x* is a hybrid node and all other nodes are internal tree nodes. If we split *v* twice by adding the elementary nodes *e*_1_ and *e*_2_ in grey, we have that (*e*_1_, *e*_2_) is an elementary path with grantor and heir equal respectively to *u* and *v*. *N* is a binary tree-child network since both parents *v* and *w* of the only hybrid node *x* have another child (1 and *y*, respectively) that is a tree node.

Given a node *u*, we can *split* it by adding a new node $\tilde{u}$, an arc $\left(\tilde{u},u\right)$, and replacing every arc (*v*, *u*) with $\left(v,\tilde{u}\right)$. If *u* is a tree node, then $\tilde{u}$ is an elementary node whose heir is *u*, and the elimination of $\tilde{u}$ recovers the original network. The successive splitting (say *k* times) of a tree node *u* generates an elementary path formed by *k* nodes, whose heir is *u*, and whose elimination recovers the original network. Fig 2 illustrates the definitions given in this section.

## Results

### Reduction of networks

The goal of this subsection is to define a reduction procedure on BTC networks that can be applied to any such network, and producing a BTC network with one leaf less. By successive application of this procedure, any BTC network can thus be reduced to the trivial network with a single leaf.

We start by associating to each leaf *ℓ* a path whose removal will produce the desired reduction (up to elementary paths).

**Fig 3:**
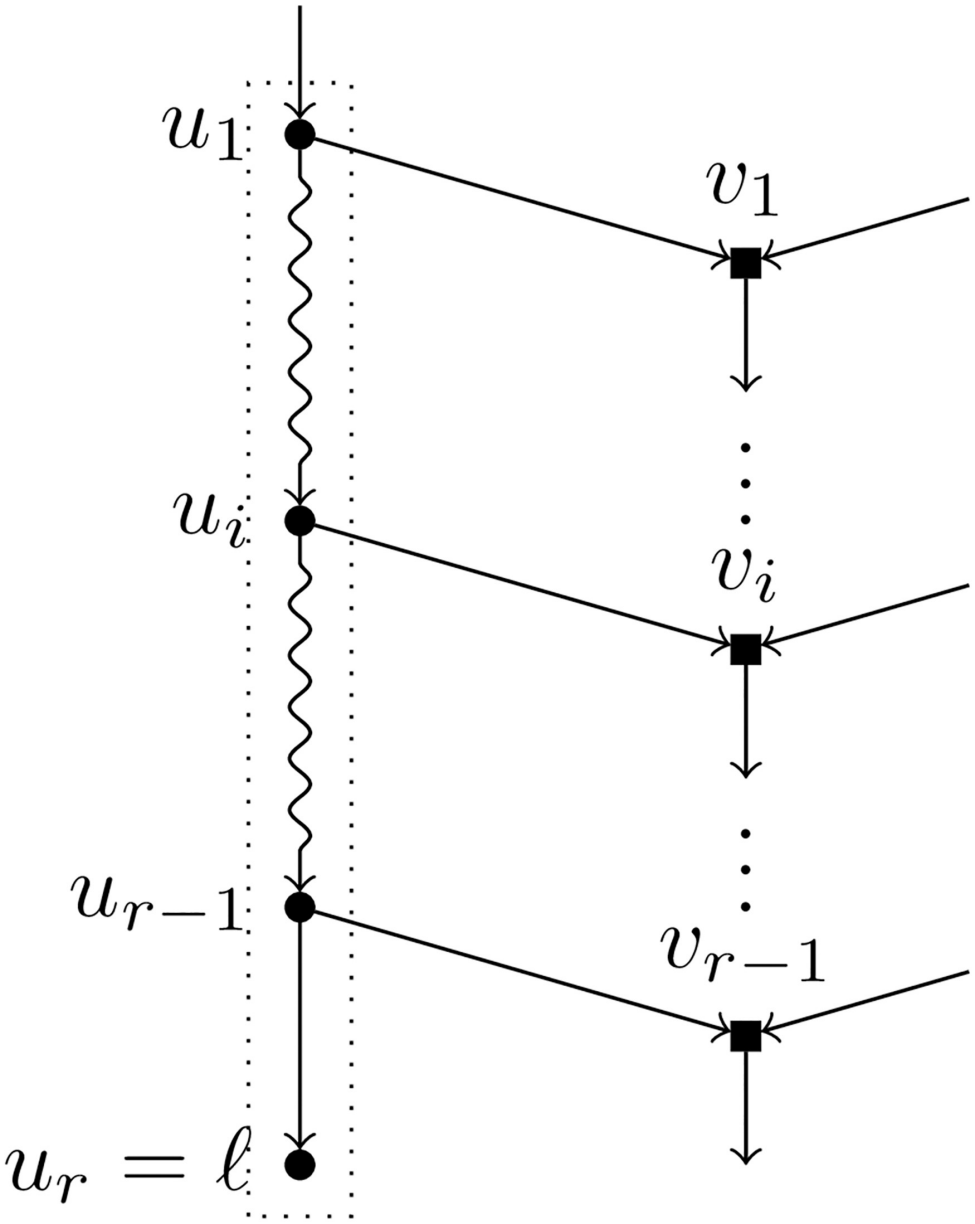
A pre-TH-path for the leaf *ℓ*. Tree nodes are represented by circles and hybrid nodes by squares; snake arrows represent paths. The path inside the dotted box is a pre-TH-path for *ℓ*.

Let *ℓ* be a leaf of a BTC network *N*. A *pre-TH-path* for *ℓ* is a path *u*_1_, …, *u*_*r*_ = *ℓ* such that (see Fig 3):

1. Each node *u*_*i*_ in the path is a tree node.
2. For each *i* = 1, …, *r* − 1, the child of *u*_*i*_ different from *u*_*i*+1_, denoted by *v*_*i*_, is a hybrid node.
3. For each *i* ≠ *j*, we have that *v*_*i*_ ≠ *v*_*j*_.

**Fig 4:**
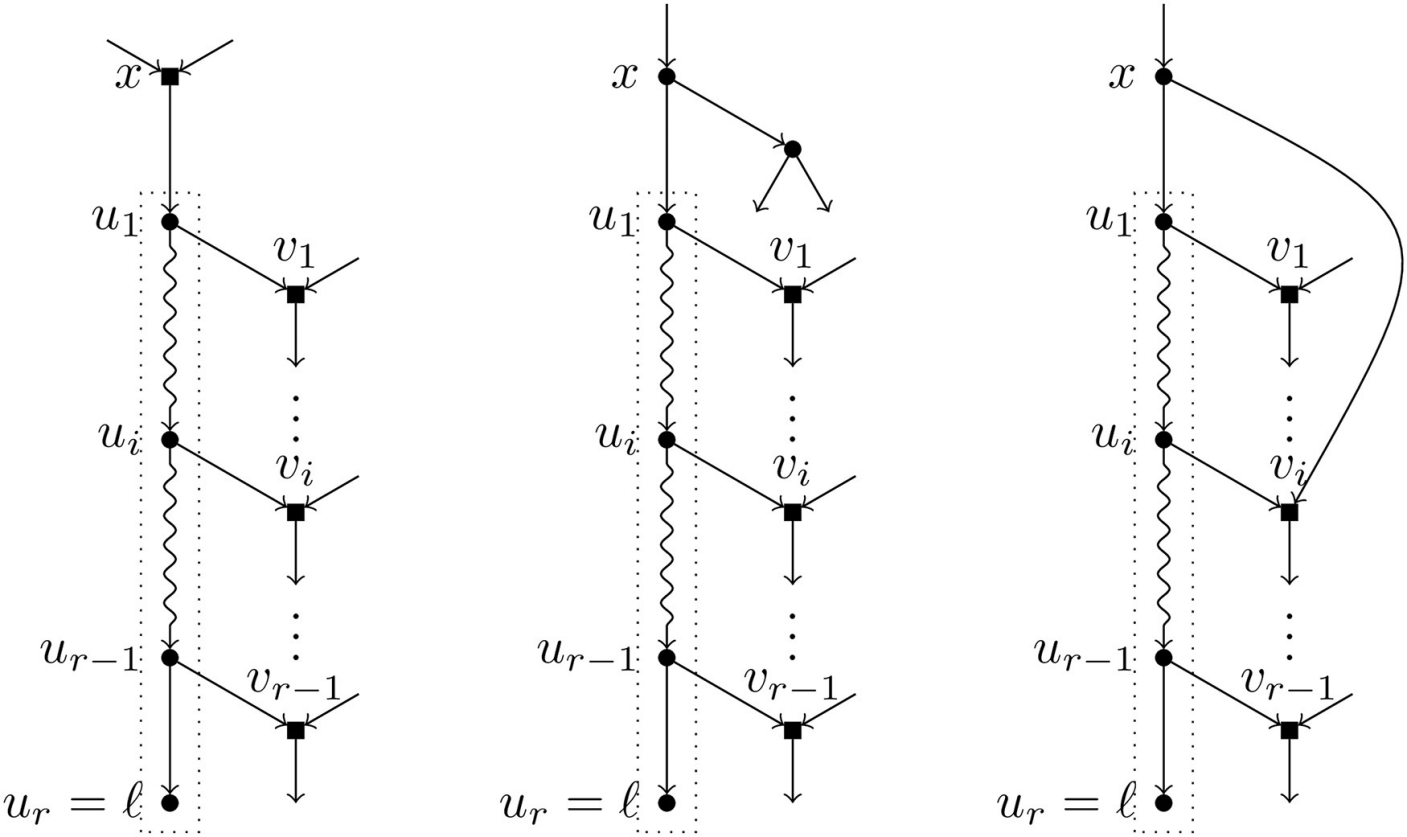
The different possibilities for the TH-path of a leaf *ℓ*. Depiction of the conditions (2), (3) and (4), respectively, under which a pre-TH-path cannot be extended, making the path inside the dotted box a TH-path for *ℓ*.

A *TH-path* is a maximal pre-TH-path, i.e. a pre-TH-path that cannot be further extended. Note that, since all nodes in a pre-TH-path *p* are tree nodes, if *p* can be extended by prepending one node, then this extension is unique. Hence, starting with the trivial pre-TH-path formed by the leaf *ℓ* alone, and extending it by prepending the parent of the first node in the path as many times as possible, we obtain a TH-path that is unique by construction. Let *u*_1_, …, *u*_*r*_ = *ℓ* be a TH-path; different possibilities may arise that make it maximal: (1) *u*_1_ is the root of *N*; (2) the parent of *u*_1_, call it *x*, is a hybrid node; (3) *x* is a tree node whose both children are tree nodes; (4) *x* is a parent of *v*_*i*_ for some *i* ∈ [*r* − 1]. We shall see in Lemma 1 that the first case cannot hold; the other three possibilities are depicted in Fig 4.

For each leaf *ℓ*, we denote by TH(*ℓ*) its single TH-path and by TH(*ℓ*)_1_ the first node of this path. Note that we allow the case *r* = 1. In this case, if we are not in a trivial BTC network (i.e. a network consisting of a single node), the parent of *ℓ* is either a hybrid node, or a tree node whose two children are tree nodes.

**Lemma 1**. *Let N be a non-trivial BTC network and let ℓ be any of its leaves. Then*, TH(*ℓ*)_1_ *cannot be the root of N*.

**Fig 5:**
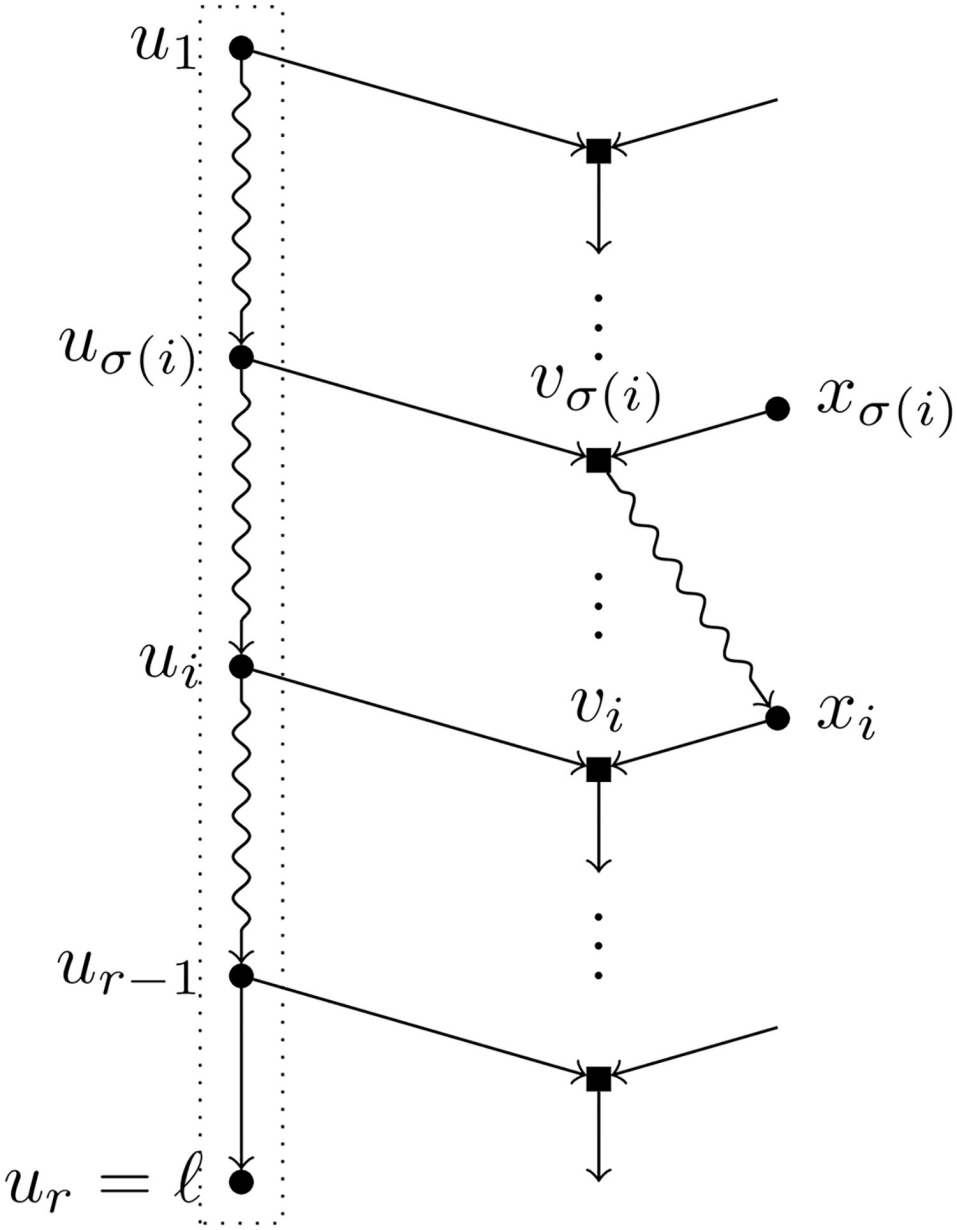
The first node in a TH-path cannot be the root. Illustration of the nodes involved in the proof of Lemma 1.

*Proof*. Let *u*_1_, …, *u*_*r*_ = *ℓ* be the path TH(*ℓ*) and assume for the sake of contradiction that *u*_1_ is the root of *N*. For each *i* = 1, …, *r* − 1, let *v*_*i*_ be the hybrid node that is a child of *u*_*i*_ and *x*_*i*_ the parent of *v*_*i*_ different from *u*_*i*_ (see Fig 5); recall that *x*_*i*_ does not belong to TH(*ℓ*) by the definition of a pre-TH-path. Since *u*_1_ is the root of *N*, every node of *N* either belongs to the path TH(*ℓ*) or is descendant of a node in {*v*_*i*_ ∣ *i* ∈ [*r* − 1]}. In particular, for each *i* ∈ [*r* − 1], there exists some *σ*(*i*) ∈ [*r* − 1] such that *x*_*i*_ is descendant of *v*_*σ*(*i*)_, and since this node is descendant of *x*_*σ*(*i*)_, *x*_*i*_ is descendant of *x*_*σ*(*i*)_. Hence, starting with *x*_1_ we get a sequence *x*_1_, *x*_*σ*(1)_, *x*_*σ*(*σ*(1))_, … where each node in the sequence is a descendant of the following one. Since there is a finite number of nodes, at some point we find a repeated node, which means that *N* contains a cycle and hence we have a contradiction.■

We say that a leaf *ℓ* is of *type* *T* (resp. of *type* *H*) if the parent of TH(*ℓ*)_1_ is a tree node (resp. a hybrid node). If *ℓ* is of type *H*, we indicate by $\bar{\mathrm{TH}}\left(\ell \right)$ the path obtained by prepending to TH(*ℓ*) the parent of TH(*ℓ*)_1_. For convenience, we let $\bar{\mathrm{TH}}\left(\ell \right)=\mathrm{TH}\left(\ell \right)$ if *ℓ* is of type *T*.

**Fig 6:**
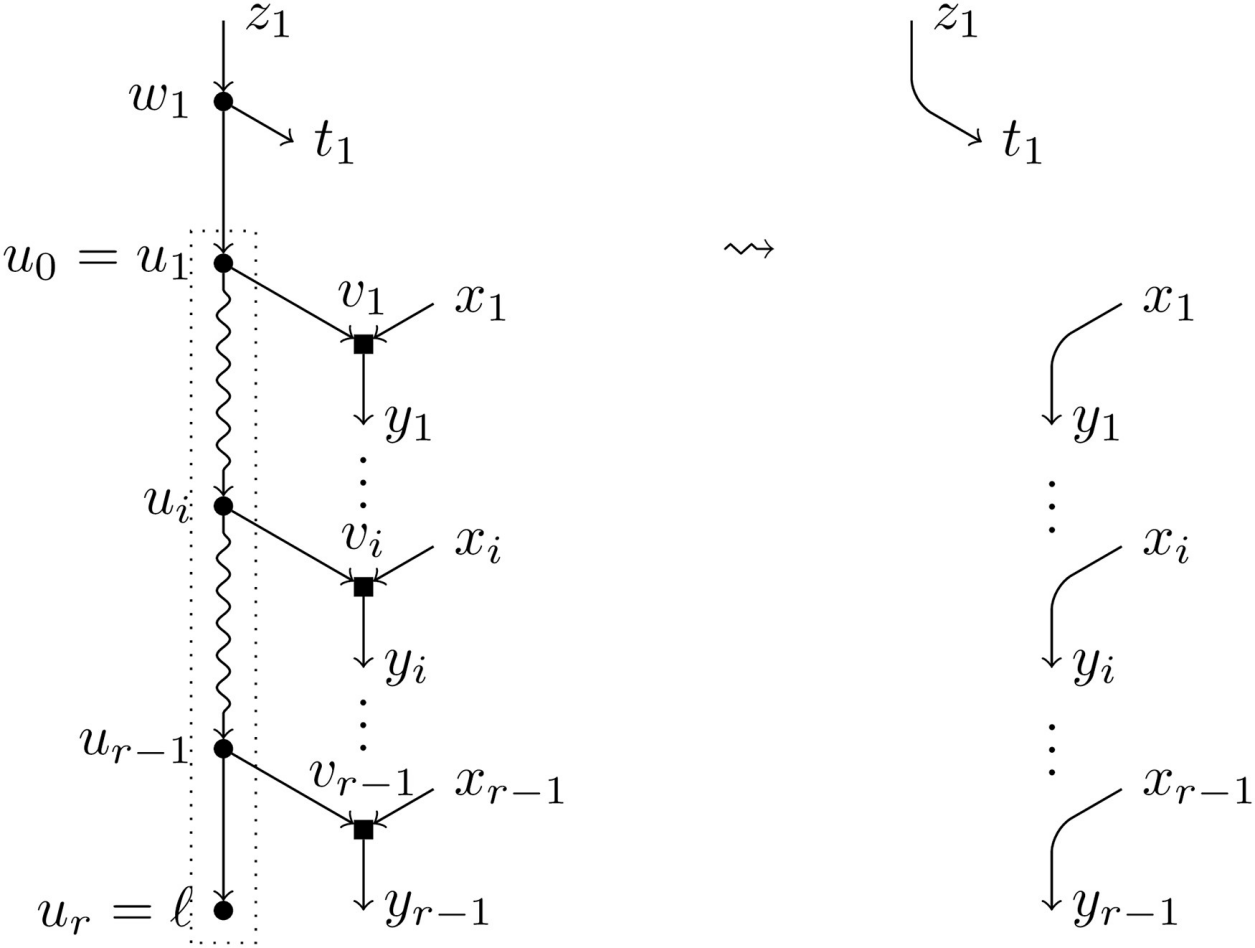
Reduction operation of type *T*. Depiction of the reduction operation *T*(*N*, *ℓ*). The nodes inside the dotted box form $\bar{\mathrm{TH}}\left(\ell \right)$ and will be removed, which will create elementary nodes that will be substituted by arcs.

**Fig 7:**
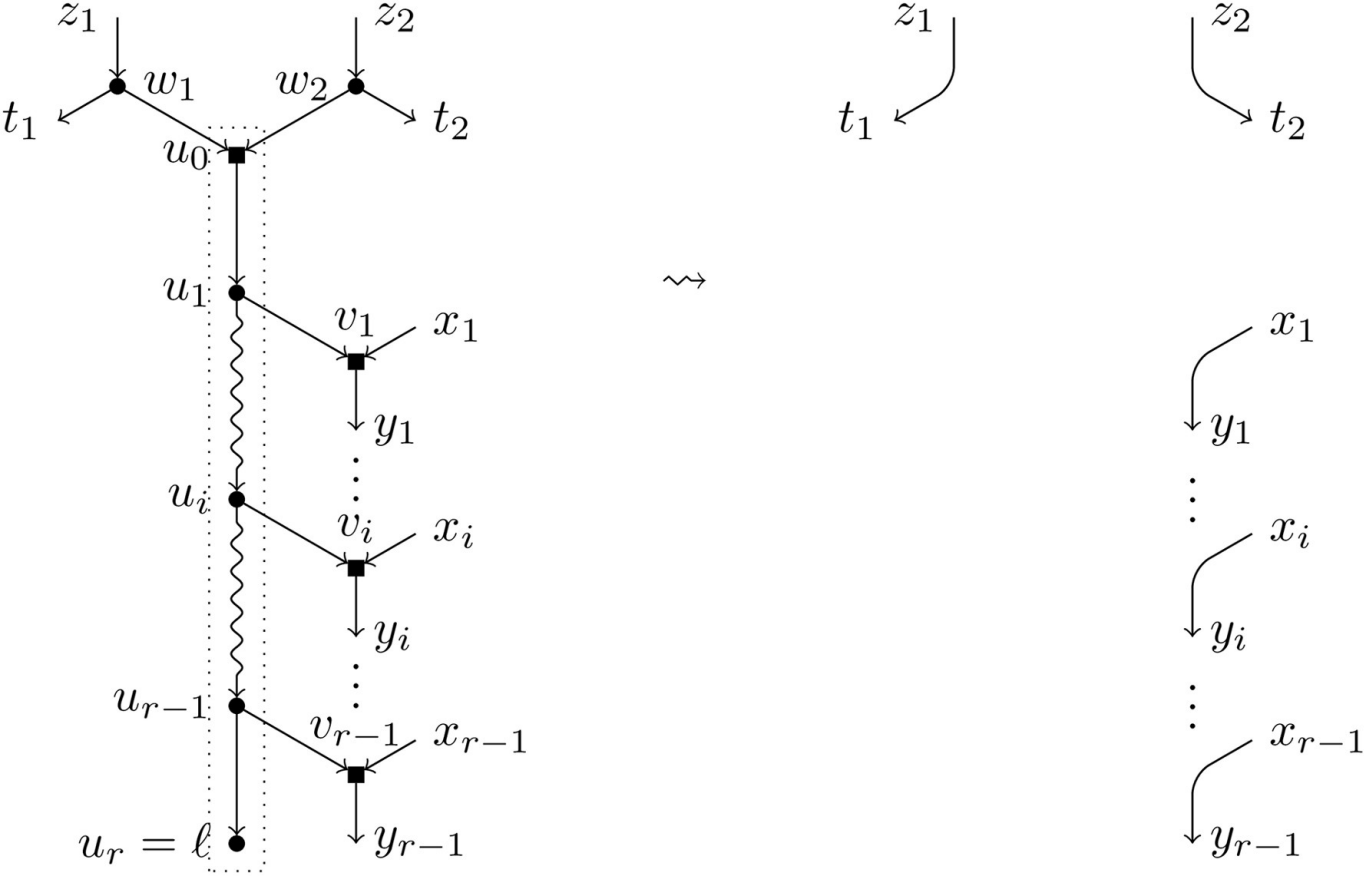
Reduction operation of type *H*. Depiction of the reduction operation *H*(*N*, *ℓ*). The nodes inside the dotted box form $\bar{\mathrm{TH}}\left(\ell \right)$ and will be removed, which will create elementary nodes that will be substituted by arcs.

**Definition 1**. Let *ℓ* be a leaf in a BTC network *N*. We define the *reduction of N with respect to ℓ* as the result of the following procedure (see Figs 6 and 7):

1. Delete all nodes in $\bar{\mathrm{TH}}\left(\ell \right)$ (together with any arc incident on them).
2. Eliminate all elementary nodes.

We indicate this reduction by *R*(*N*, *ℓ*). If we want to emphasize the type of the deleted leaf, we indicate the reduction by *T*(*N*, *ℓ*) and say it is a *T*-reduction if *ℓ* is of type *T*, or by *H*(*N*, *ℓ*) and say that it is a *H*-reduction if *ℓ* is of type *H*.

To ease of reading, we shall introduce some notations that will be used hereafter and are also illustrated in Figs 6 and 7:

**Definition 2**. Let *u*_1_, …, *u*_*r*_ = *ℓ* be the path TH(*ℓ*) and let *u*_0_ be the first node in $\bar{\mathrm{TH}}\left(\ell \right)$. For each *i* ∈ [*r* − 1], *v*_*i*_ is the hybrid child of *u*_*i*_, *x*_*i*_ the parent of *v*_*i*_ different from *u*_*i*_, and *y*_*i*_ the single child of *v*_*i*_. The parent(s) of *u*_0_ is *w*_1_ (are *w*_1_, *w*_2_); the node *w*_*j*_ is always a tree node, *z*_*j*_ is its parent (if it exists, since *w*_*j*_ could be the root of *N*), and *t*_*j*_ its child different from *u*_0_, where *j* = 1 for *T*-reductions and *j* ∈ [2] for *H*-reductions.

**Remark 1**. Since *N* is tree-child, the nodes *y*_*i*_ are always tree nodes, and so are *t*_1_ and *t*_2_ in case of an *H*-reduction. In case of a *T*-reduction, by definition of a TH-path, *t*_1_ is either a tree node or coincides with one of the hybrid nodes *v*_*i*_. Also, the removal of the arcs of the form (*u*_*i*_, *v*_*i*_) and (*w*_*j*_, *u*_0_) makes nodes *v*_*i*_ and *w*_*j*_ elementary in $N\backslash \bar{\mathrm{TH}}\left(\ell \right)$, where *i* ∈ [*r* − 1], and *j* = 1 for *T*-reductions and *j* ∈ [2] for *H*-reductions. Since no other arc is removed, no other node can be elementary. In order to find the heirs of nodes *v*_*i*_ and *w*_*j*_, we must analyse under which circumstances two of these elementary nodes are adjacent in $N\backslash \bar{\mathrm{TH}}\left(\ell \right)$.

**Fig 8:**
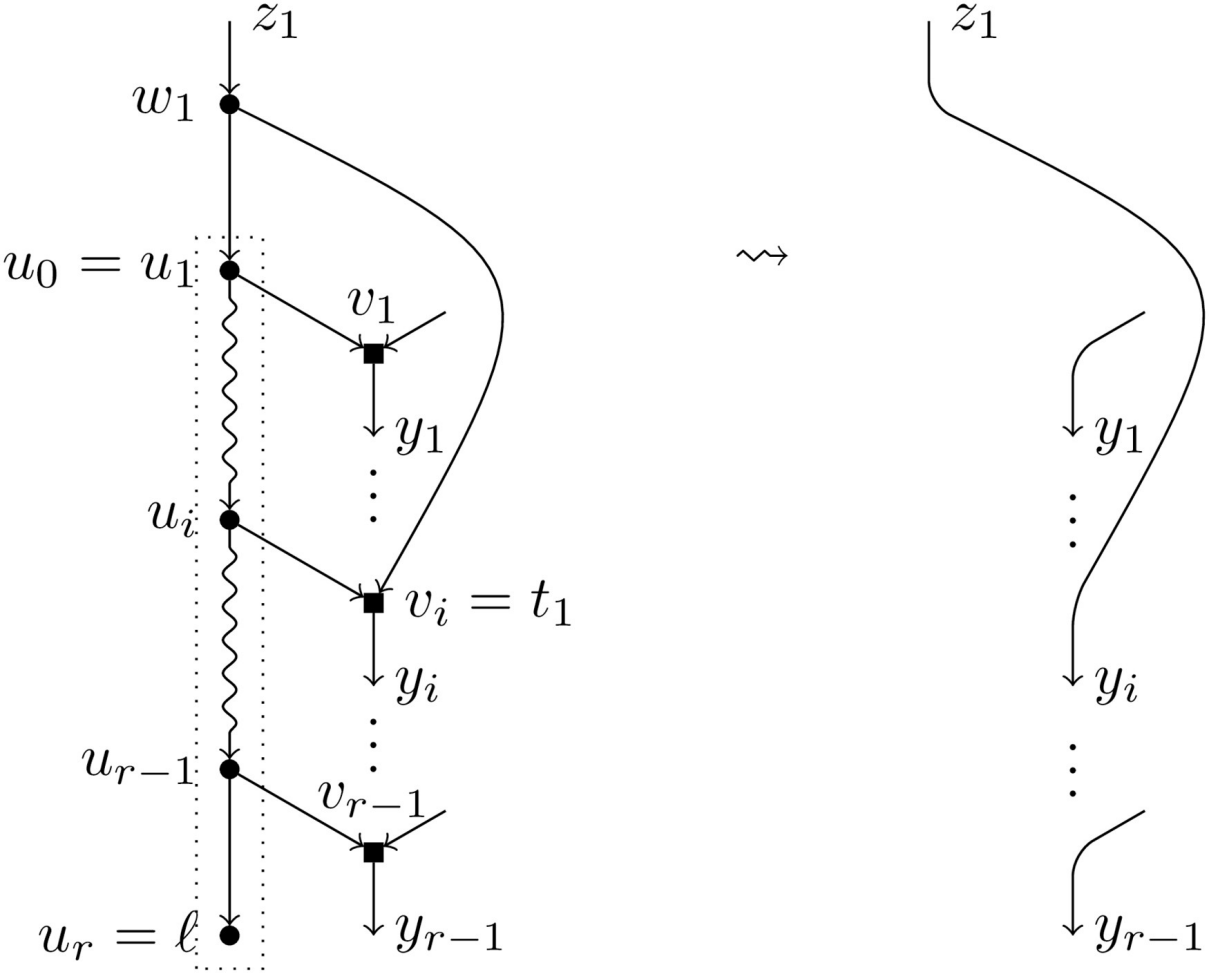
Reduction operation of type *T* (particular case). Particular case of the reduction operation of type *T* when *t*_1_ = *v*_*i*_ for some *i* ∈ [*r* − 1].

**Fig 9:**
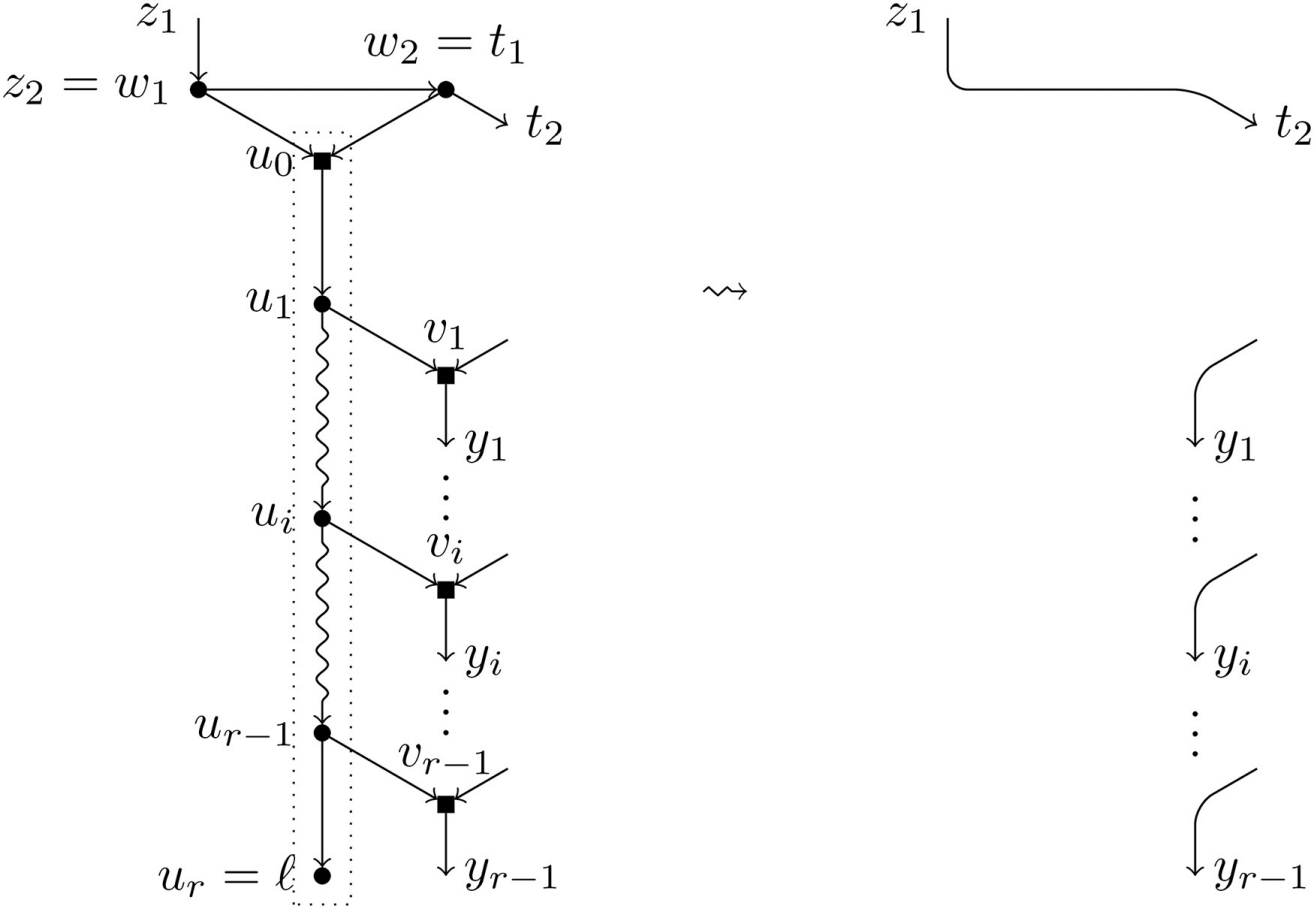
Reduction operation of type *H* (particular case). Particular case of the reduction operation of type *H* when *w*_1_ and *w*_2_ are linked by an arc.

1. If we had that two nodes *v*_*i*_ and *v*_*j*_ were connected by an arc in $N\backslash \bar{\mathrm{TH}}\left(\ell \right)$, then the single child of a hybrid node in *N* would be also a hybrid. This contradicts the fact that *N* is tree-child.
2. The existence of an arc (*v*_*i*_, *w*_*j*_) would imply the existence of a cycle in *N*, which is impossible.
3. Consider now the case of an arc (*w*_*j*_, *v*_*i*_). In case of an *H*-reduction, it would imply that both children of *w*_*j*_ are hybrid nodes, which is impossible. However, such an arc can be present in a *T*-reduction: when *t*_1_ is equal to *v*_*i*_. In this last case, *w*_1_ and *v*_*i*_ form an elementary path in $N\backslash \bar{\mathrm{TH}}\left(\ell \right)$ and their common heir is *y*_*i*_ (see Fig 8).
4. Finally, in case of an *H*-reduction, it can exist an arc between *w*_1_ and *w*_2_, say that the arc is (*w*_1_, *w*_2_) (which implies, *t*_1_ = *w*_2_, *z*_2_ = *w*_1_). In this case, *w*_1_ and *w*_2_ form an elementary path in $N\backslash \bar{\mathrm{TH}}\left(\ell \right)$ and their common heir is *t*_2_ (see Fig 9).

In all other cases, the elementary nodes *v*_*i*_ and *w*_*j*_ are isolated, and their respective heirs are *y*_*i*_ and *t*_*j*_.

We study now what we call the *recovering data* of a reduction. This information will be used in the next subsection to recover the original network from its reduction.

**Definition 3**. The *recovering data* of the reduction *N*′ = *R*(*N*, *ℓ*) is the pair (*S*_1_, *S*_2_), where:

- *S*_1_ is the multiset of the nodes of *N*′ that are heirs of the nodes *w*_*j*_. The cardinality of *S*_1_ (as a multiset) is either 1 or 2, depending on the type of the reduction, and will be denoted by |*S*_1_|.
- *S*_2_ is the tuple (*y*_1_, …, *y*_*r*−1_) of nodes of *N*′, which are the heirs of the nodes *v*_*i*_. This tuple could be empty, corresponding to the case *r* = 1.

We introduce now a set of conditions on multisets and tuples of nodes, and prove that the recovering data associated to any of the defined reductions satisfies them.

**Definition 4**. Given a BTC network *N*′ and a pair (*S*_1_, *S*_2_) with

- *S*_1_ a multiset of tree nodes of *N*′,
- *S*_2_ = (*y*_1_, …, *y*_*r*−1_), with *r* ≥ 1, a (potentially empty) tuple of *r* − 1 tree nodes of *N*′,

consider the following set of conditions:

1 For every *i*, *j* ∈ [*r* − 1] with *i* ≠ *j*, the nodes *y*_*i*_ and *y*_*j*_ are different, and if they are siblings, then *y*_*i*_ ∈ *S*_1_ or *y*_*j*_ ∈ *S*_1_.
2 For every *i* ∈ [*r* − 1], if *y*_*i*_ is the child of a hybrid node or has a hybrid sibling, then *y*_*i*_ ∈ *S*_1_.
3 No node in *S*_1_ is a proper descendant of any node in *S*_2_.
4T |*S*_1_| = 1.
4H |*S*_2_| = 2 and no node of *S*_1_ appears in *S*_2_.

We say that (*S*_1_, *S*_2_) is *T-feasible* if it satisfies conditions 1, 2, 3, and 4T, and *H-feasible* if it satisfies conditions 1, 2, 3, and 4H. Finally, we say that (*S*_1_, *S*_2_) is *feasible* if it is either *T*-feasible or *H*-feasible.

**Proposition 2**. *Let N*′ = *T*(*N*, *ℓ*) *be a T-reduction of a BTC network N. Then, its recovering data* ({*τ*_1_}, (*y*_1_, …, *y*_*r*−1_)) *is T-feasible*.

*Proof*. First, note that, by Remark 1, all nodes in ({*τ*_1_}, (*y*_1_, …, *y*_*r*−1_)) are tree nodes and that Condition 4T holds trivially. Note also that *τ*_1_ is equal to *y*_*i*_ if *t*_1_ = *v*_*i*_, or to *t*_1_ if this node is different from all the nodes *v*_*i*_. We now prove that Conditions 1, 2 and 3 hold:

1. If *y*_*i*_ = *y*_*j*_, then in *N* we have *v*_*i*_ = *v*_*j*_, which is impossible by definition of TH-path. If *y*_*i*_ and *y*_*j*_ are siblings in *N*′ but none of these nodes is equal to *τ*_1_, then *v*_*i*_ and *v*_*j*_ are siblings in *N*, which implies that their common parent has two hybrid children, which is impossible in a BTC network.
2. If *y*_*i*_ is the child in *N*′ of a hybrid node and *τ*_1_ ≠ *y*_*i*_, then in *N* we have that *v*_*i*_, which is a hybrid node, is the child of a hybrid node, which is impossible in a tree-child network. Analogously, if *y*_*i*_ has a sibling in *N*′ which is a hybrid node, and *y*_*i*_ ≠ *τ*_1_, then in *N* we have that *v*_*i*_ is sibling of another hybrid node, which is again impossible.
3. The existence of a non-trivial path in *N*′ from *y*_*i*_ to *τ*_1_ would, by construction, imply the existence of a path from *y*_*i*_ to *w*_1_ in *N*. Since there exists also a path in *N* from *w*_1_ to *y*_*i*_, this would contradict the fact that *N* is a DAG.■

**Proposition 3**. *Let N*′ = *H*(*N*, *ℓ*) *be an H-reduction of a BTC network N. Then, its recovering data* ({*τ*_1_, *τ*_2_}, (*y*_1_, …, *y*_*r*−1_)) *is H-feasible*.

*Proof*. Again we have, by Remark 1, that all nodes in the recovering data are tree nodes. Additionally, by the same remark, we have that |*S*_1_| = 2 –and hence the first part of Condition 4H holds– and if (*w*_1_, *w*_2_) is an arc of *N*, then *S*_1_ = {*t*_2_, *t*_2_}, otherwise *S*_1_ = {*t*_1_, *t*_2_} with *t*_1_ ≠ *t*_2_. Note that Condition 3 implies that Conditions 1 and 2 can be simplified as follows: for all *i*, *j* ∈ [*r* − 1] with *i* ≠ *j*, *y*_*i*_ and *y*_*j*_ are neither equal nor siblings, and for all *i* ∈ [*r* − 1], *y*_*i*_ is neither the child nor the sibling of a hybrid node.

Conditions 1 and Conditions 2 and 3 in their simplified form follow using the same arguments as in the previous proposition. As for the condition 4H, the nodes *τ*_1_ and *τ*_2_ are different from the nodes *y*_*i*_ since the parents of *τ*_1_ and *τ*_2_ in *N* are tree nodes, while the parent of each of the nodes *y*_*i*_ is hybrid.■

The following proposition is the main result of this subsection, since it shows that the reduction that we have defined, when applied to a BTC network, gives another BTC network with one leaf less. Hence, successive applications of these reductions reduce any BTC network to the trivial BTC network.

**Proposition 4**. *Let N be a BTC network over X and ℓ one of its leaves. Then, R*(*N*, *ℓ*) *is a BTC network over X* \ {*ℓ*}.

*Proof*. First, it is easy to see that, since no new path is added, the resulting directed graph is still acyclic.

Then, we need to check that *R*(*N*, *ℓ*) is binary. To do so, we start noting that every node in $N\backslash \bar{\mathrm{TH}}\left(\ell \right)$ is either a tree node, a hybrid node, or an elementary node. Indeed, the removal of $\bar{\mathrm{TH}}\left(\ell \right)$ (Phase 1 of Definition 1) only affects the nodes adjacent to this path, that is the nodes *v*_*i*_ and *w*_*i*_, which, as shown in Remark 1, become elementary. The elimination of all elementary nodes (Phase 2 of Definition 1) does not affect the indegree and outdegree of any other node, apart when the root *ρ* of $N\backslash \bar{\mathrm{TH}}\left(\ell \right)$ is elementary. In such a case, the heir of *ρ* becomes the new root. Hence, *R*(*N*, *ℓ*) is binary and rooted.

Note also that the set of leaves of *R*(*N*, *ℓ*) is *X* \ {*ℓ*}, since in $N\backslash \bar{\mathrm{TH}}\left(\ell \right)$ no node becomes a leaf and the only leaf that is removed is *ℓ*.

Finally, we need to prove that *R*(*N*, *ℓ*) is tree-child. Note that, from what we have just said about how the reduction affects indegrees and outdegrees of the nodes that persist in the network, it follows that each hybrid node of *R*(*N*, *ℓ*) is also a hybrid node of *N*, and that its parents in *R*(*N*, *ℓ*) are the same as in *N*. It follows that no node in *R*(*N*, *ℓ*) can have that all its children are hybrid, since this would imply that *N* is not tree-child, a contradiction.■

**Corollary 5**. *Let* $N\in {BTC}_{n}$ *be a BTC network over* [*n*]. *Let N*_*n*_ = *N and define recursively N*_*i*_ = *R*(*N*_*i*+1_, *i* + 1) *for each i* = *n* − 1, *n* − 2, …, 1. *Then, N*_*i*_ *is a BTC network over* [*i*]. *In particular, N*_1_ *is the trivial BTC network with its single node labeled by* 1.

We finish this subsection with the computation of the number of tree nodes and hybrid nodes that the reduced network has, both in terms of the original network and of the reduction operation that has been applied. But before, we give an absolute bound on the number of these nodes in terms of the number of leaves.

**Lemma 6**. *Let N be BTC network over* [*n*] *with t tree nodes and h hybrid nodes. Then t* − *h* = 2*n* − 1, *h* ≤ *n* − 1 *and t* ≤ 3*n* − 2.

*Proof*. The equality *t* − *h* = 2*n* − 1 follows easily from the handshake lemma taking into account the number of roots, internal tree nodes, leaves and hybrid nodes in *N*, and their respective indegrees and outdegrees. The inequality *h* ≤ *n* − 1 is shown in Proposition 1 in [9], and the last inequality is a simple consequence of the equality and the inequality already proved.■

**Proposition 7**. *Let N be a BTC network and ℓ one of its leaves, and N*′ = *R*(*N*, *ℓ*). *Let t, h (resp. t′, h′) be the number of tree nodes and hybrid nodes of N (resp. of N′). Then*

$$\begin{array}{c} t^{\prime }=t-|\bar{\mathrm{TH}}\left(\ell \right)|-1,\;h^{\prime }=h-\left|\bar{\mathrm{TH}}\left(\ell \right)\right|+1, \end{array}$$

*where* $|\bar{\mathrm{TH}}\left(\ell \right)|$ *is the number of nodes in* $\bar{\mathrm{TH}}\left(\ell \right)$.

*Proof*. Since the number of tree nodes and hybrid nodes are linked by the equality in Lemma 6, it is enough to prove that $h^{\prime }=h-\left|\bar{\mathrm{TH}}\left(\ell \right)\right|+1$. From the discussion in Remark 1, it is straightforward to see that the number of hybrid nodes in *N* that are not in *N*′ is *r* − 1 if *ℓ* is of kind *T*, and *r* otherwise. Hence, in both cases we have $h^{\prime }=h-\left(\right|\bar{\mathrm{TH}}\left(\ell \right)\left|-1\right)$ and the result follows.■

### Generation of networks

In this subsection, we consider the problem of how to revert the reductions defined in the previous subsection, taking as input the reduced network and its recovering data. This will allow us to define a procedure that, starting with the trivial BTC network with one leaf, generates all the BTC networks with any number of leaves in a unique way.

We start by defining two augmentation procedures that take as input a BTC network and a feasible pair, and produce a BTC network with one leaf more.

**Fig 10:**
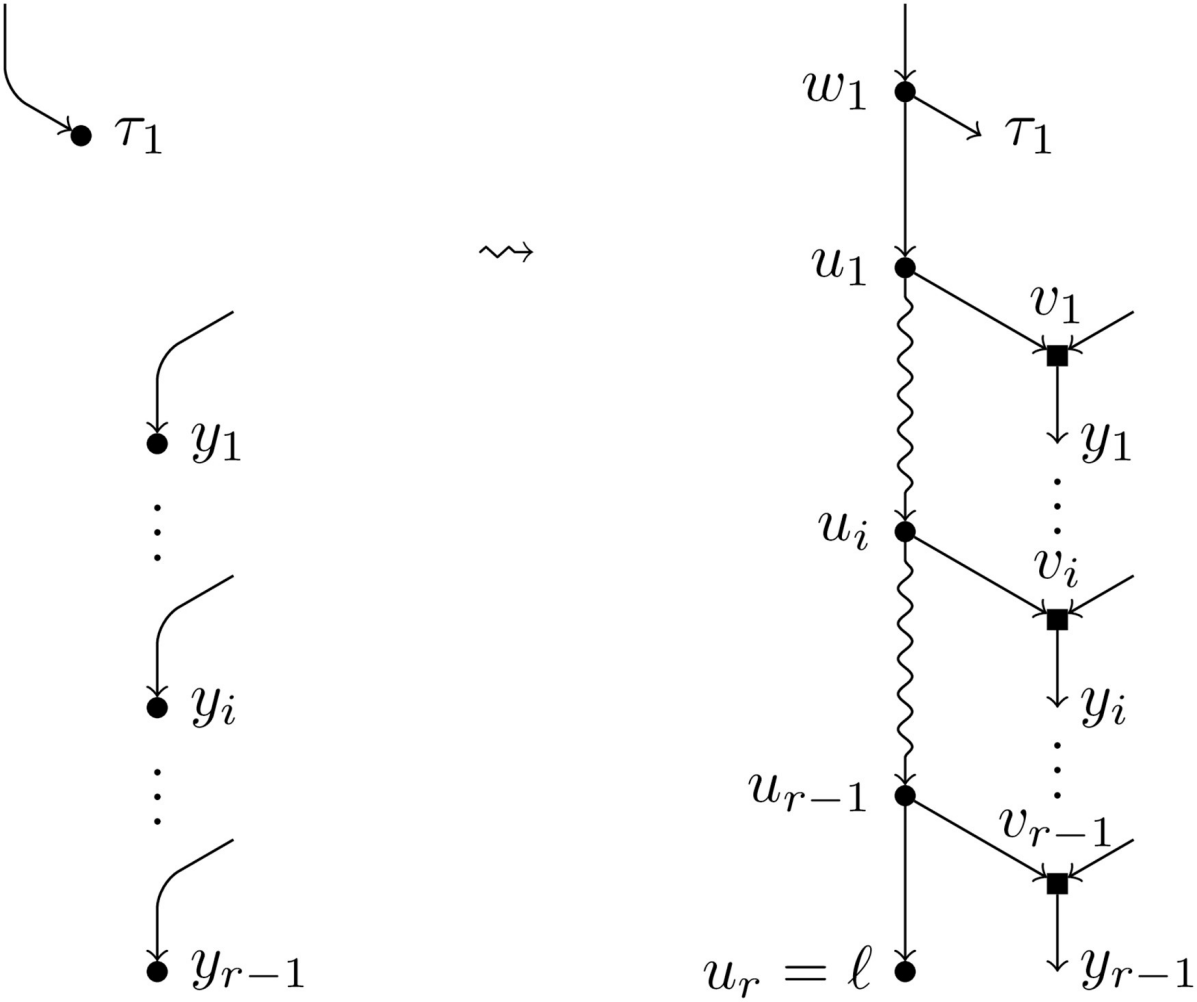
Augmentation operation of type *T*. Depiction of the augmentation operation *T*^−1^(*N*, *ℓ*; {*τ*_1_}, (*y*_1_, …, *y*_*r*−1_)) when *τ*_1_ ≠ *y*_*i*_ for all *i* ∈ [*r* − 1].

**Definition 5**. Let *N* be a BTC network over *X*, *ℓ* a label not in *X*, and ({*τ*_1_}, (*y*_1_, …, *y*_*r*−1_)) a *T*-feasible pair. We apply the following operations to *N* (see Fig 10):

1. Create a path of new nodes *u*_1_, …, *u*_*r*_.
2. Split the node *τ*_1_ creating one elementary node *w*_1_ and add an arc (*w*_1_, *u*_1_).
3. For each node *y*_*i*_, split it introducing one elementary node *v*_*i*_ and add an arc (*u*_*i*_, *v*_*i*_).
4. Label the node *u*_*r*_ by *ℓ*.

We denote by *T*^−1^(*N*, *ℓ*; {*τ*_1_}, (*y*_1_, …, *y*_*r*−1_)) the resulting network and say that it has been obtained by an *augmentation operation of type* *T*.

**Fig 11:**
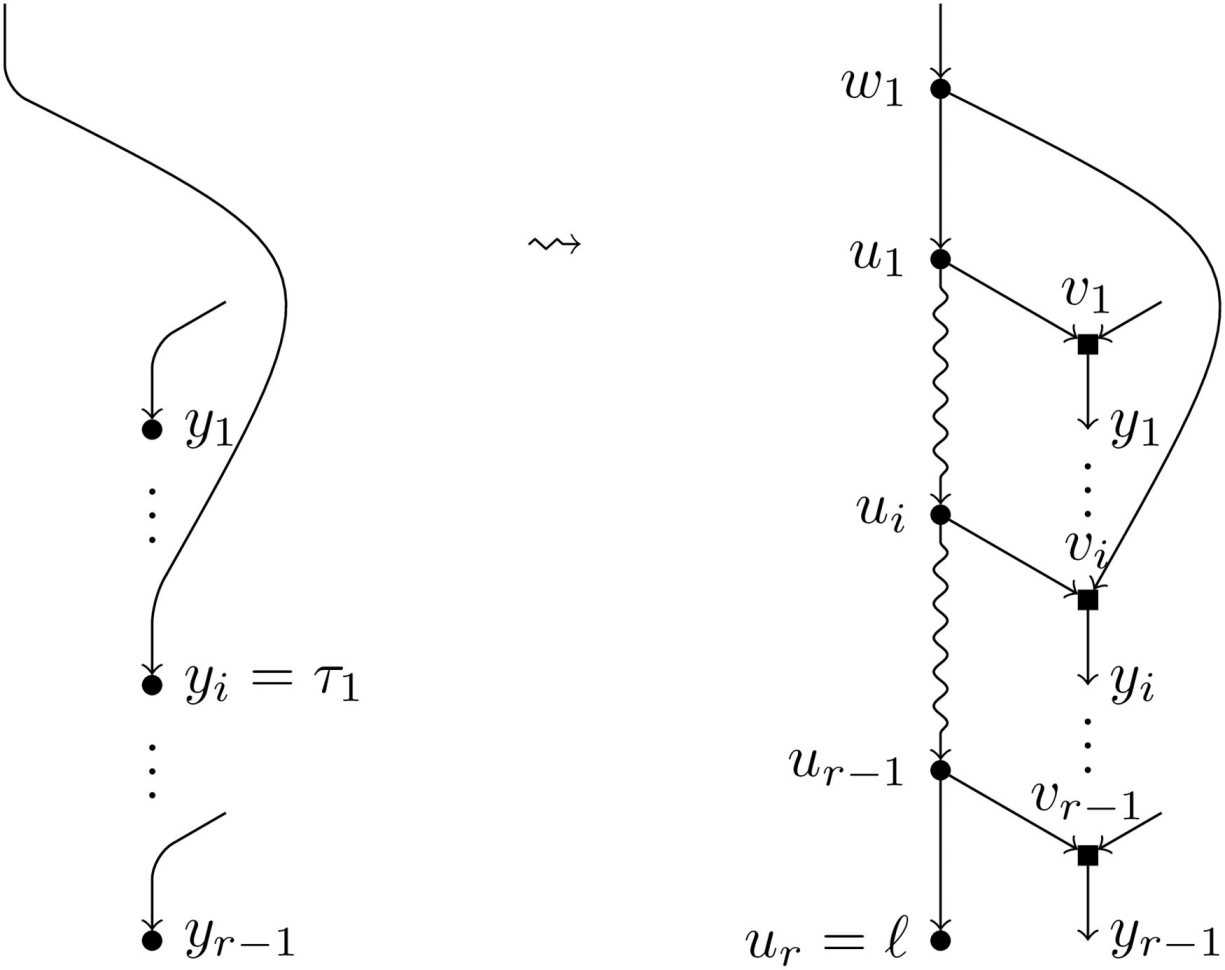
Augmentation operation of type *T* (particular case). Depiction of the augmentation operation *T*^−1^(*N*, *ℓ*; {*τ*_1_}, (*y*_1_, …, *y*_*r*−1_)) when *τ*_1_ = *y*_*i*_.

Note that the order in which steps 2 and 3 are done is relevant in the case that *τ*_1_ = *y*_*i*_ for some *i* ∈ [*r* − 1]. In such a case, two nodes *w*_1_ and *v*_*i*_ are created, linked by an arc (*w*_1_, *v*_*i*_) (see Fig 11).

**Proposition 8**. *Using the notations of Definition 5, the network*

$$\begin{array}{c} \tilde{N}=T^{-1}\left(N,\ell ;\left\{\tau_{1}\right\},\left(y_{1},\ldots ,y_{r-1}\right)\right) \end{array}$$

*is a BTC network over X* ∪ {*ℓ*}. *Moreover, if N has h hybrid nodes, then* $\tilde{N}$ *has h* + *r* − 1 *hybrid nodes*.

*Proof*. We first check that the resulting directed graph is acyclic. Let us assume that $\tilde{N}$ contains a cycle. If we define *U*_1_ = {*u*_1_, …, *u*_*r*_} and $U_{2}=V\left(\tilde{N}\right)\backslash U_{1}$, we have that the only arcs connecting *U*_1_ with *U*_2_ are (*u*_*i*_, *v*_*i*_) (with *i* = 1, …, *r* − 1), and (*w*_1_, *u*_1_) is the only arc connecting *U*_2_ with *U*_1_. The cycle can be contained neither inside *U*_1_, since these nodes are linked by a single path, nor inside *U*_2_, since otherwise *N* would contain a cycle. Hence, the cycle must contain at least the arc (*w*_1_, *u*_1_) and an arc (*u*_*i*_, *v*_*i*_). This implies the existence of a path from *v*_*i*_ to *w*_1_ visiting only nodes in *U*_2_, which in turn means that *N* contains a path from *y*_*i*_ to *τ*_1_, against Condition 3 of Definition 4.

Note that the nodes in *U*_1_ are tree nodes by construction. Also by construction, the node *w*_1_ is a tree node, the nodes *v*_*i*_ are hybrid nodes and *u*_*r*_ is a leaf which is labelled with *ℓ*. Finally, the other nodes keep the same degrees they had in *N* and hence $\tilde{N}$ is a binary phylogenetic network over *X* ∪ {*ℓ*} with *h* + *r* − 1 hybrid nodes.

Since *N* is tree-child, in order to check that $\tilde{N}$ is also tree-child, we only need to check the newly added hybrid nodes, which are the parents of the nodes *v*_*i*_.

Let us first consider the case that *τ*_1_ ≠ *y*_*i*_ for all *i* ∈ [*r* − 1]. For each node *v*_*i*_, its parents are *u*_*i*_ and the parent *x*_*i*_ of *y*_*i*_ in *N*. The node *u*_*i*_ is by construction a tree node whose other child is *u*_*i*+1_, which, in turn, is a tree node. Since *τ*_1_ ≠ *y*_*i*_, by Condition 2 of Definition 4, *y*_*i*_ can have neither a hybrid parent nor a hybrid sibling, and it cannot be a sibling of any other node *y*_*j*_ with *j* ∈ [*r* − 1]. This latter restriction implies that *y*_*i*_ has the same sibling ${\tilde{x}}_{i}$ in *N* and $\tilde{N}$. Thus both *x*_*i*_ and ${\tilde{x}}_{i}$ are not hybrid nodes, and the network is tree-child.

Let us now consider the case that *τ*_1_ = *y*_*i*_ for a single choice of *i* ∈ [*r* − 1]. The hybrid node *v*_*i*_ in $\tilde{N}$ has as parents the nodes *w*_1_ and *u*_*i*_, and these two nodes have as respective children *u*_1_ and *u*_*i*+1_, which are tree nodes. For each other node *v*_*j*_ with *j* ≠ *i* and such that *y*_*j*_ is a not sibling of *y*_*i*_, the same argument as in the previous case proves that both parents of *v*_*j*_ have a tree child. If *y*_*j*_ is a sibling of *y*_*i*_, it is easy to see that the parent of *v*_*j*_ is still tree-child since it has *w*_1_ as child.■

**Fig 12:**
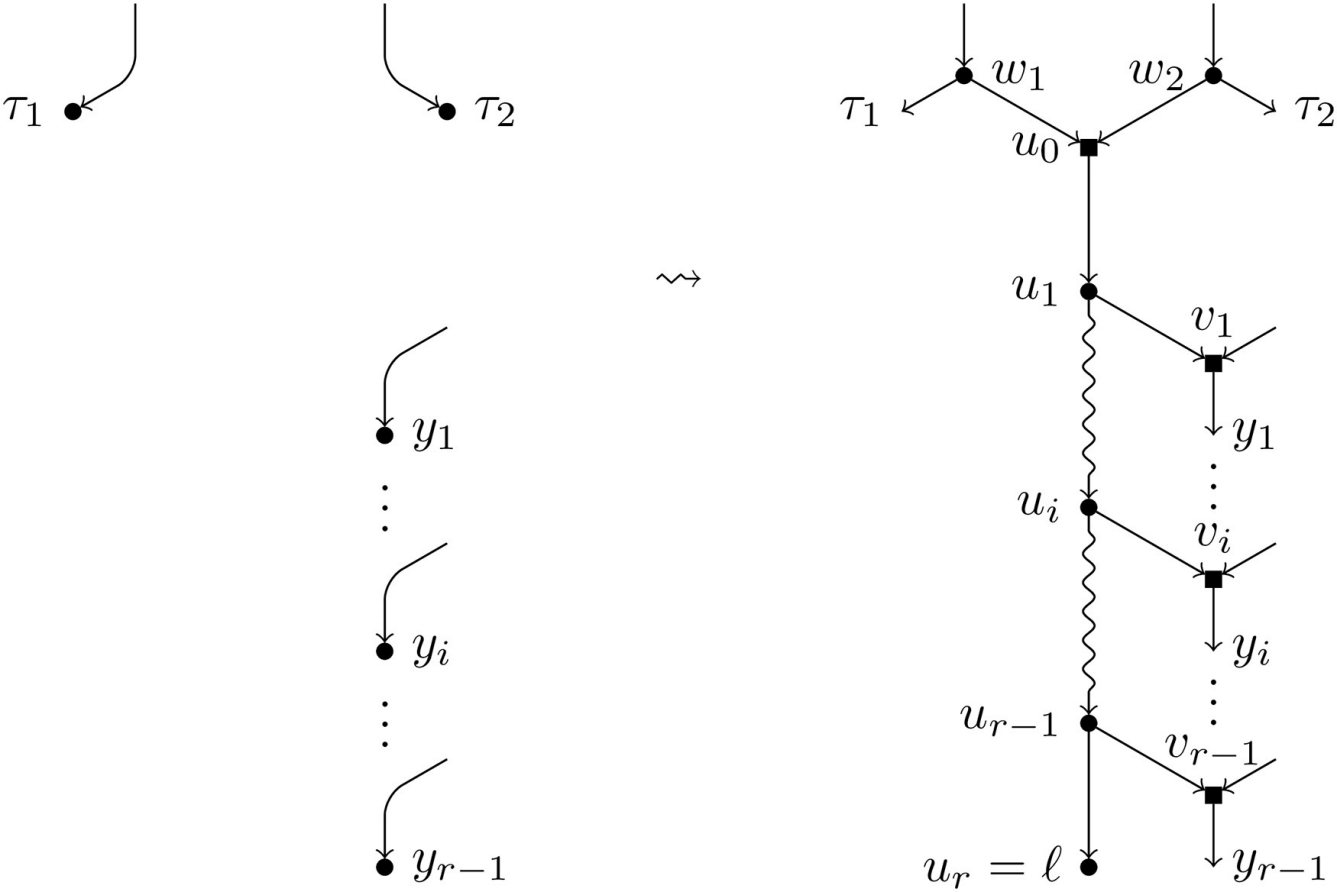
Augmentation operation of type *H*. Depiction of the augmentation operation *H*^−1^(*N*, *ℓ*; {*τ*_1_, *τ*_2_}, (*y*_1_, …, *y*_*r*−1_)) when *τ*_1_ ≠ *τ*_2_.

**Fig 13:**
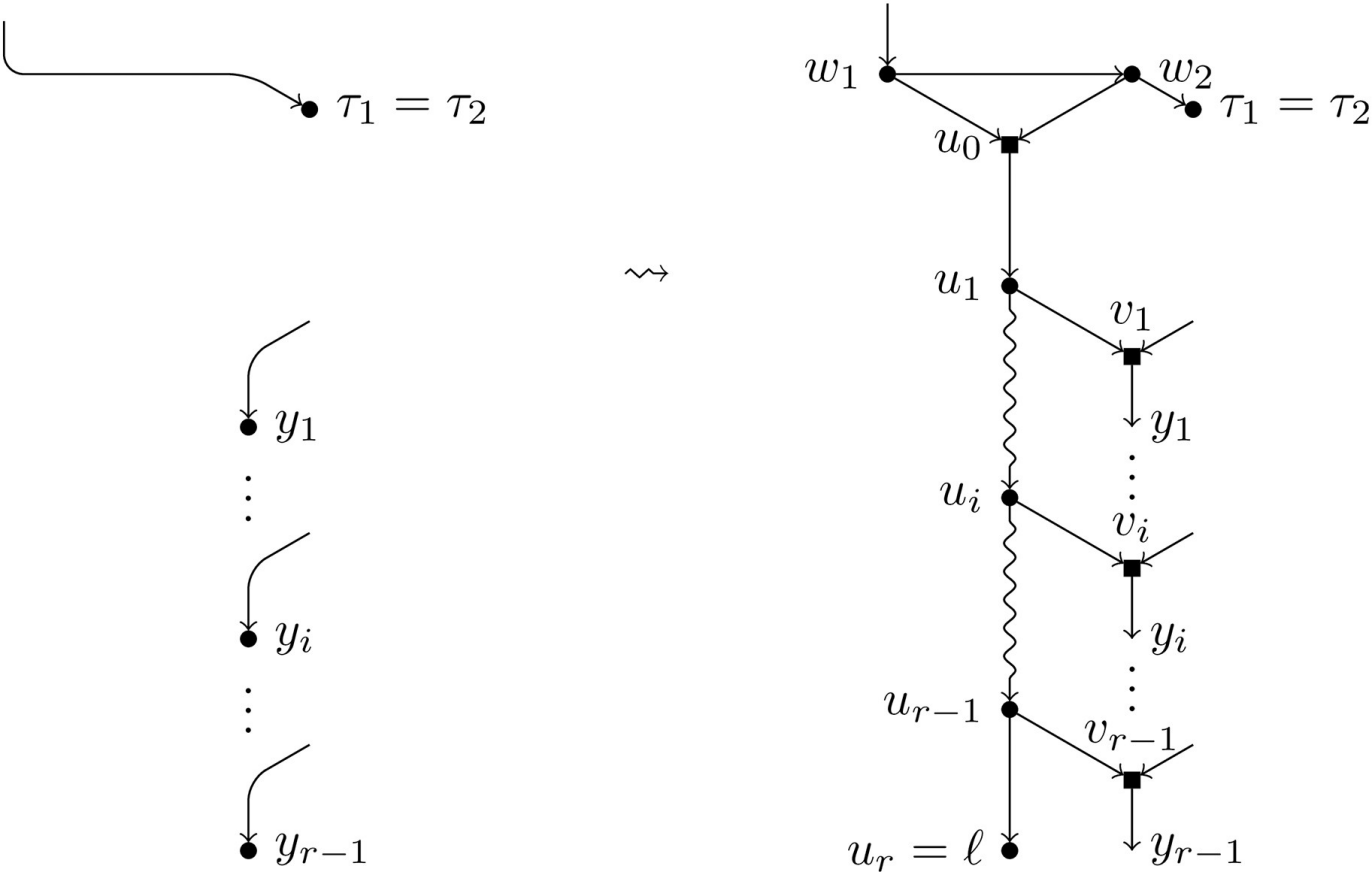
Augmentation operation of type *H* (particular case). Depiction of the augmentation operation *H*^−1^(*N*, *ℓ*; {*τ*_1_, *τ*_2_}, (*y*_1_, …, *y*_*r*−1_)) when *τ*_1_ = *τ*_2_.

**Definition 6**. Let *N* be a BTC network over *X*, *ℓ* a label not in *X*, and ({*τ*_1_, *τ*_2_}, (*y*_1_, …, *y*_*r*−1_) a *H*-feasible pair. We apply the following operations to *N* (see Fig 12):

1. Create a path of new nodes *u*_0_, *u*_1_, …, *u*_*r*_.
2. Split each of the nodes *τ*_*i*_ introducing one elementary node *w*_*i*_ and add an arc from *w*_*i*_ to *u*_0_. Note that, if *τ*_1_ = *τ*_2_, two consecutive elementary nodes must be created (see Fig 13 for this case).
3. For each node *y*_*i*_, split it introducing one elementary node *v*_*i*_ and add an arc (*u*_*i*_, *v*_*i*_).
4. Label the node *u*_*r*_ by *ℓ*.

We denote by *H*^−1^(*N*, *ℓ*;{*τ*_1_, *τ*_2_}, (*y*_1_, …, *y*_*r*−1_)) the resulting network and say that it has been obtained by an augmentation operation of type *H*.

**Proposition 9**. *Using the notations of Definition 6, the network*

$$\begin{array}{c} \tilde{N}=H^{-1}\left(N,\ell ;\left\{\tau_{1},\tau_{2}\right\},\left(y_{1},\ldots ,y_{r-1}\right)\right) \end{array}$$

*is a BTC network over X* ∪ {*ℓ*}. *If N has h hybrid nodes, then* $\tilde{N}$ *has h* + *r hybrid nodes*.

*Proof*. The proof is completely analogous to that of Proposition 8, taking into account that one extra hybrid node is created.■

Given a BTC network over *X*, a label *ℓ* ∉ *X* and a feasible pair (*S*_1_, *S*_2_), in order to unify notations we define the augmented network *R*^−1^(*N*, *ℓ*; *S*_1_, *S*_2_) as *T*^−1^(*N*, *ℓ*; *S*_1_, *S*_2_), if |*S*_1_| = 1, and as *H*^−1^(*N*, *ℓ*; *S*_1_, *S*_2_), if |*S*_1_| = 2. Also, we shall generically say that the *offspring* of a BTC network is the set of networks that can be obtained from it by means of augmentation operations.

Our next goal is to prove that different augmentation operations applied to a same BTC network or different BTC networks over the same set of taxa provide different networks. We start with the case of different networks.

**Proposition 10**. *Let* ${\tilde{N}}_{1}$ *and* ${\tilde{N}}_{2}$ *be two BTC networks, both obtained by one augmentation operation applied to two non-isomorphic BTC networks N*_1_ *and N*_2_ *over the same set of taxa X. Then* ${\tilde{N}}_{1}$ *and* ${\tilde{N}}_{2}$ *are not isomorphic*.

*Proof*. If ${\tilde{N}}_{1}$ and ${\tilde{N}}_{2}$ have different sets of labels, then it is clear that they are not isomorphic. We can therefore assume that both augmentation operations introduced the same new leaf *ℓ*. Suppose that ${\tilde{N}}_{1}≃{\tilde{N}}_{2}$. Then $R\left({\tilde{N}}_{1},\ell \right)≃R\left(N_{2},\ell \right)$. Now, from the definitions of the reductions and augmentations it is straightforward to check that $R\left({\tilde{N}}_{i},\ell \right)=N_{i}$ and we get that *N*_1_ ≃ *N*_2_, a contradiction.■

We treat now the case of applying different augmentation operations to the same BTC network. But first, we give a technical lemma that will be useful in the proof of the proposition.

**Lemma 11**. *Let N be a BTC network. Then, the identity is the only automorphism (as a leaf-labeled directed graph) of N*.

*Proof*. Let *ϕ* be any automorphism of *N*. Since *ϕ* is an automorphism of directed graphs and sends each leaf to itself, it follows that *μ*(*u*) = *μ*(*ϕ*(*u*)) for each node *u* of *N*, where *μ*(*u*) is the *μ*-vector of *u* as defined in [9]. Then, by [9, Lemma 5c], it follows that *u* and *ϕ*(*u*) are either equal, or one of them is the single child of the other one; according to our definition of BTC networks, this last possibility implies that one of them is a hybrid node and the other one is a tree node, which is impossible if *ϕ* is an automorphism. Hence *ϕ*(*u*) = *u* for every node *u*.■

**Proposition 12**. *Let* ${\tilde{N}}_{1}$ *and* ${\tilde{N}}_{2}$ *be two BTC networks, both obtained by one augmentation operation applied to the same BTC network N. If either the kinds of operation or the feasible pairs used to construct* ${\tilde{N}}_{1}$ *and* ${\tilde{N}}_{2}$ *are different, then* ${\tilde{N}}_{1}$ *and* ${\tilde{N}}_{2}$ *are not isomorphic*.

*Proof*. Let us assume that ${\tilde{N}}_{1}$ and ${\tilde{N}}_{2}$ are isomorphic. Then, it is clear that they have the same set of labels, and exactly one of them, say *ℓ*, is not a label of *N*. Since ${\tilde{N}}_{1}$ and ${\tilde{N}}_{2}$ are isomorphic, the kind of *ℓ* is the same in both networks, which implies that the kind of augmentation operations used to construct ${\tilde{N}}_{1}$ and ${\tilde{N}}_{2}$ are the same. Also, since ${\tilde{N}}_{1}$ and ${\tilde{N}}_{2}$ are isomorphic, the nodes in the respective recovering data of the reductions $R({\tilde{N}}_{i},\ell )$ must be linked by an isomorphism of phylogenetic networks. Therefore, and since by Lemma 11 BTC networks do not have a nontrivial automorphism, the respective recovering data must be equal.■

The following proposition shows that the reduction procedure defined in the previous subsection can be reverted using the augmentation operations presented in this subsection.

**Proposition 13**. *Let N be a BTC network and ℓ a leaf of N. Let N*′ = *R*(*N*, *ℓ*), (*S*_1_, *S*_2_) *its recovering data, and* $\tilde{N}=R^{-1}\left(N^{\prime },\ell ;S_{1},S_{2}\right)$. *Then, N and* $\tilde{N}$ *are isomorphic*.

*Proof*. It is straightforward to see that the operations *T*^−1^ and *H*^−1^ reverse the effects of *T* and *H*, respectively. The only points worthy of attention correspond to the cases where the single node in *S*_1_ appears in *S*_2_ (for reductions/augmentations of type *T*) or where there is a single node in *S*_1_ with multiplicity two (for reductions/augmentations of type *H*). In the first case, the augmentation process creates two elementary nodes, *w*_1_ and *v*_*i*_, connected by an arc (*w*_1_, *v*_*i*_), which is the same situation as in *N* after the removal of the nodes in $\bar{\mathrm{TH}}\left(\ell \right)$. In the second case, two elementary nodes *τ*_1_ and *τ*_2_ are created, connected by an arc, once again the same situation as in *N* after the removal of the nodes in $\bar{\mathrm{TH}}\left(\ell \right)$.■

**Fig 14:**
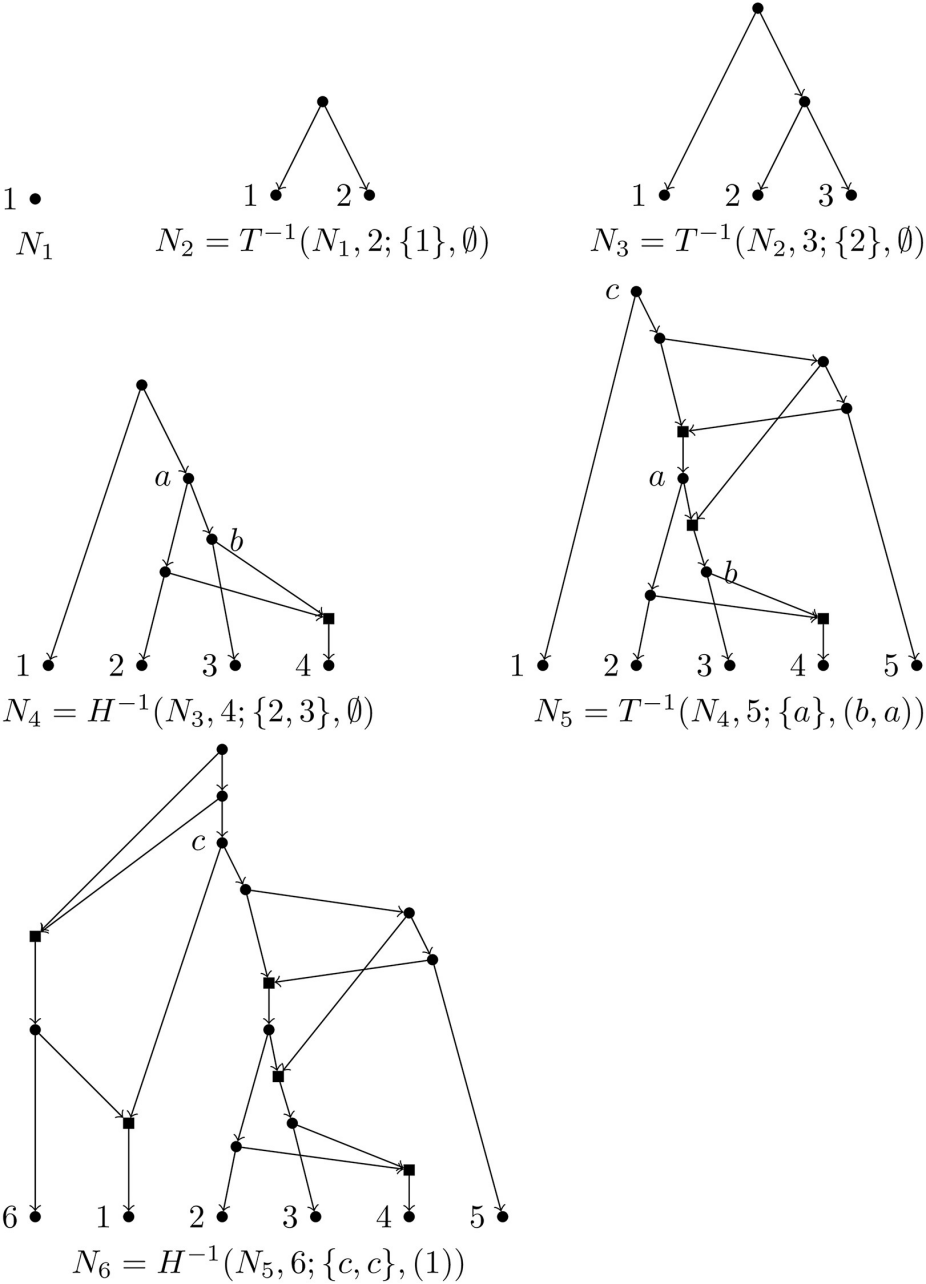
Construction of a BTC network. Example of a chain of augmentation operations that generate a BTC network.

A direct consequence of the results in this subsection is the following theorem, which can be used to generate in an effective way all BTC networks over a set of taxa. See Fig 14 for an example.

**Theorem 14**. *Let* $N\in {BTC}_{n}$ *be a BTC network over* [*n*]. *Then, N can be constructed from the trivial network in* ${BTC}_{1}$ (*with one node labeled by* 1) *by application of n* − 1 *augmentation operations, where at each step i, the leaf i* + 1 *is added. Moreover, these augmentation operations are unique*.

*Proof*. The existence is a direct consequence of Corollary 5 and Proposition 13. Unicity comes from Propositions 10 and 12.■

It should be noted that very recently, other methods to generate all BTC networks over a set of taxa have been proposed [5], but, to our knowledge, this is the first time that the networks are generated with unicity. In previous attempts, an isomorphism check was needed after the generation phase.

### Bounding the number of networks

In this subsection, we shall first give bounds for the number of BTC networks that can be obtained from a given one by means of augmentation operations. This will be done by bounding the number of feasible pairs in such a network. Then, we shall find bounds for the number of BTC networks with a fixed number *n* of leaves.

Let *N* be a BTC network over [*n*] with *h* hybrid nodes. From Lemma 6 we know that it has *t* = 2*n* + *h* − 1 tree nodes, and that *h* ≤ *n* − 1 and *t* ≤ 3*n* − 2. In the following, we shall show how to compute the number of pairs (*S*_1_, *S*_2_) satisfying all conditions of Definition 4, except for Condition 3, via an auxiliary problem. Note that this will only give an upper bound for the number of networks, since the pairs we find can produce networks with cycles.

#### An auxiliary problem

Let *P*(*N*, *k*) be the set of tuples of length *k* of tree nodes of *N* such that (1) no pair of them are equal or siblings, and (2) none of them has a hybrid parent or sibling. We indicate the number of such tuples as *p*(*N*, *k*) = |*P*(*N*, *k*)|, and since this number will only depend on *n*, *h* and *k*, we indicate it also by *p*(*n*, *h*, *k*). We consider the problem of computing *p*(*n*, *h*, *k*).

We compute first how many tree nodes are there that neither have a hybrid parent nor a hybrid sibling. Since the single child of a hybrid node must be a tree node, there are *h* tree nodes that have a hybrid parent. Note that each hybrid node has two siblings that must be tree nodes; also, a tree node cannot be sibling of two different hybrid nodes; hence, there are 2*h* tree nodes that have a hybrid sibling. Since there cannot be a tree node having the two properties (if it has a hybrid parent, then it does not have any hybrid sibling), there are 3*h* tree nodes that are either a child or a sibling of a hybrid node. Then, the number of tree nodes that neither have a hybrid parent nor a hybrid sibling is *t* − 3*h* = 2*n* − 2*h* − 1. Note that this set of nodes is composed by the root of the network and pairs of tree nodes that are siblings.

Consider now the problem of counting the number of tuples (*y*_1_, …, *y*_*k*_) in this set that are neither equal nor siblings. We distinguish two cases:

- If none of the nodes *y*_*i*_ is the root of *N*, we start having 2*n* − 2*h* − 2 choices for *y*_1_, and at each stage the number of choices decreases by two units. Hence, the number of choices is
  $$\begin{array}{c} \begin{array}{cc} p_{0}\left(n,h,k\right) & =(2n-2h-2)(2n-2h-4)\cdots (2n-2h-2k)\\ & =2^{k}\left(n-h-1\right)\left(n-h-2\right)\cdots \left(n-h-k\right)\\ & =2^{k}\frac{(n-h-1)!}{(n-h-k-1)!}. \end{array} \end{array}$$
- If one of the nodes *y*_*i*_ is the root of *N*, then the process of constructing an element in *P*(*N*, *k*) can be described as first choosing at which position *i* one puts the root, and then filling in the remaining *k* − 1 positions with a tuple of the set *P*(*N*, *k* − 1) such that none of the nodes is the root (which is what we have just computed). Hence, the number of possibilities is
  $$\begin{array}{c} p_{1}\left(n,h,k\right)=k2^{k-1}\frac{(n-h-1)!}{(n-h-k)!}. \end{array}$$

Then we get that

$$\begin{array}{c} \begin{array}{cc} p(n,h,k) & =p_{0}\left(n,h,k\right)+p_{1}\left(n,h,k\right)\\ & =2^{k}\frac{(n-h-1)!}{(n-h-k-1)!}+k2^{k-1}\frac{(n-h-1)!}{(n-h-k)!} \end{array} \end{array}$$

#### Counting pairs satisfying conditions 1, 2 and 4H

Consider pairs (*S*_1_, *S*_2_) satisfying Conditions 1, 2 and 4H. Recall that, since condition 4H implies that *S*_1_ and *S*_2_ cannot have elements in common, Conditions 1 and 2 are simplified: no pair of nodes in *S*_2_ can be siblings and none of them can either be the child of a hybrid node or have a hybrid sibling. Hence, the problem is equivalent to finding a tuple (*y*_1_, …, *y*_*r*−1_) in *P*(*N*, *r* − 1) and then either a tree node *τ*_1_ or an unordered pair {*τ*_1_, *τ*_2_} of different tree nodes, in either case disjoint from those in (*y*_1_, …, *y*_*r*−1_). Once the tuple (*y*_1_, …, *y*_*r*−1_) is fixed, the number of tree nodes available for choosing *τ*_1_ and *τ*_2_ is *t* − *r* + 1 = 2*n* + *h* − *r*. Hence, the number of possible pairs is

$$\begin{array}{c} F_{H}\left(n,h,r-1\right)=F_{H,1}\left(n,h,r-1\right)+F_{H,2}\left(n,h,r-1\right), \end{array}$$

where

$$\begin{array}{c} \begin{array}{cc} F_{H,1}\left(n,h,r-1\right) & =p(n,h,r-1)\cdot (2n+h-r),\\ F_{H,2}\left(n,h,r-1\right) & =p\left(n,h,r-1\right)\cdot \frac{1}{2}\left(2n+h-r\right)\left(2n+h-r-1\right). \end{array} \end{array}$$

#### Counting pairs satisfying conditions 1, 2 and 4T

The problem now is to count the ways of choosing *S*_1_ = {*τ*_1_} and a tuple *S*_2_ = (*y*_1_, …, *y*_*r*−1_) satisfying Conditions 1, 2 and 4T. Now *τ*_1_ can appear in *S*_2_, and different possibilities arise, since it allows that one of the nodes in *S*_2_ has a sibling in *S*_2_, or that it has a hybrid parent or sibling. We consider, thus, these different possibilities:

- *τ*_1_ ≠ *y*_*i*_ (for all *i*): This case is very similar to the one considered in the previous paragraph, specifically the case where only a single node *τ*_1_ had to be taken. The number of possible pairs is
  $$\begin{array}{c} F_{T,1}\left(n,h,r-1\right)=p\left(n,h,r-1\right)\cdot \left(2n+h-r\right). \end{array}$$
- *τ*_1_ = *y*_*i*_ is a child or a sibling of a hybrid node: Choosing one of these pairs is equivalent to first choosing the position *i*, then filling the other *r* − 2 positions with a tuple in *P*(*N*, *r* − 2), and then choosing a node that is a child or sibling of a hybrid node to be put in the position *i*. The number of ways to do this procedure is
  $$\begin{array}{c} F_{T,2}\left(n,h,r-1\right)=p\left(n,h,r-2\right)\cdot \left(r-1\right)\cdot 3h, \end{array}$$
  since each hybrid node has a single child and two siblings, and none of these 3*h* nodes appears twice, associated to two different hybrid nodes.
- *τ*_1_ = *y*_*i*_ is a sibling of some other node *y*_*j*_ in *S*_2_: In this case one has to choose the positions *i* and *j* where to put the pair of sibling nodes, fill the other *r* − 3 positions with a tuple in *P*(*N*, *r* − 3), choose a pair of sibling tree nodes to take as *y*_*i*_ and *y*_*j*_, and finally set *τ*_1_ = *y*_*i*_. The choice of *i* and *j* can be done in (*r* − 1)(*r* − 2) different ways. The choice of the tuple of length *r* − 3 can be done in *p*(*n*, *h*, *r* − 3) ways; *p*_1_(*n*, *h*, *r* − 3) of them contain the root of *N* (and *r* − 4 tree nodes with a sibling tree node) and *p*_0_(*n*, *h*, *r* − 3) do not contain the root (and contain *r* − 3 tree nodes with a sibling tree node). Once this is done, the number of available pairs of sibling tree nodes is *n* − *h* − 1 − (*r* − 4) = *n* − *h* − *r* + 3, if the root of *N* was chosen, or *n* − *h* − 1 − (*r* − 3) = *n* − *h* − *r* + 2 otherwise. Hence, the total number of pairs is *F*_*T*,3_(*n*, *h*, *r* − 1) = *F*_*T*,3, *A*_(*n*, *h*, *r* − 1) + *F*_*T*,3,*B*_(*n*, *h*, *r* − 1), corresponding to these two cases, with:
  $$\begin{array}{c} \begin{array}{cc} F_{T,3,A}\left(n,h,r-1\right) & =\left(r-1\right)\left(r-2\right)\cdot p_{1}\left(n,h,r-3\right)\cdot \left(2n-2h-2r+6\right),\\ F_{T,3,B}\left(n,h,r-1\right) & =\left(r-1\right)\left(r-2\right)\cdot p_{0}\left(n,h,r-3\right)\cdot \left(2n-2h-2r+4\right). \end{array} \end{array}$$
- *τ*_1_ = *y*_*i*_ but none of the previous conditions hold: In this case one only has to take a tuple in *P*(*N*, *r* − 1) and choose which of the *r* − 1 nodes to take as *τ*_1_. The number of possible pairs is then
  $$\begin{array}{c} F_{T,4}\left(n,h,r-1\right)=p\left(n,h,r-1\right)\cdot \left(r-1\right). \end{array}$$

Note that the four conditions above are mutually exclusive. Hence, the overall number of possible pairs (*S*_1_, *S*_2_) is the sum of all numbers found:

$$\begin{array}{c} F_{T}\left(n,h,r-1\right)=F_{T,1}\left(n,h,r-1\right)+F_{T,2}\left(n,h,r-1\right)+F_{T,3}\left(n,h,r-1\right)+F_{T,4}\left(n,h,r-1\right). \end{array}$$

#### Bounds for the number of networks

Each network $N\in {BTC}_{n}$ with *h* hybrid nodes, appears as augmentation *R*^−1^(*N*′, *n*, *S*_1_, *S*_2_) of a unique network $N^{\prime }\in {BTC}_{n-1}$ with *h*′ hybrid nodes, where *S*_2_ has length *r* − 1 = *h* − *h*′, if the augmentation is of type *T*, or *r* − 1 = *h* − *h*′ − 1 if it is of type *H*. If we call *B*(*n*, *h*) the number of networks in ${BTC}_{n}$ with *h* hybrid nodes, and since we have bounded the number of feasible pairs, we have that

$$\begin{array}{c} \begin{array}{cc} B(n,h) & \leq \sum_{h^{\prime }=0}^{h}B\left(n-1,h^{\prime }\right)\cdot F_{T}\left(n-1,h^{\prime },h-h^{\prime }\right)+\\ & \;+\sum_{h^{\prime }=0}^{h-1}B\left(n-1,h^{\prime }\right)\cdot F_{H}\left(n-1,h^{\prime },h-h^{\prime }-1\right) \end{array} \end{array}$$

Also, since the number of hybrid nodes in a BTC network with *n* leaves is at most *n* − 1, we have that

$$\begin{array}{c} |{BTC}_{n}|=\sum_{h=0}^{n-1}B\left(n,h\right), \end{array}$$

and the expression above allows us to compute a bound for this number of networks. See Subsection Computational experiments for an experiment with these bounds.

The asymptotic formula $|{BTC}_{n}|=2^{2n\log n+O(n)}$ is given in [19], and both our experimental results in Subsection Computational experiments for *n* ≤ 8 and the bounds that we have computed for *n* ≤ 10 are coherent with this expression. However, the problem of finding a closed expression for the asymptotic behaviour of our bounds is still open.

### An application to phylogenetic reconstruction

Several models of reticulate evolution on biological sequences have been proposed in the last decades, for example the displayed trees model [25], an extension of the multispecies coalescent (MSC) to phylogenetic networks [26] and the ancestral recombination graph model –ARG for short [27]– to only name a few. The associated problems are difficult to solve and big efforts have been done by the community to provide practitioners with fast algorithms.

Suppose we are given a BTC network *N* over a set of OTUs *X*, where each tree node is associated with a word in an alphabet (for instance a DNA sequence) *s*(*u*) ∈ Σ*. The pair (*N*, *s*) can, for example, be the outcome of an ML search in the space of BTC networks given an alignment over *X*. Now, suppose we are given a new sequence and we want to update *N* to include it, ensuring that the resulting network is still BTC. We may want to do this, for instance, to update the network without redoing the whole ML search, or in a phylogenetic placement perspective (for example, we want to know where to place a given strain of a virus in *N*), or even because we use a heuristic algorithm that reconstructs a network by adding one sequence at the time.

We assume that a model of evolution is given, and we assume that we can compute the following probabilities:

1. Given *s*, *s*′ ∈ Σ*, *P*_*S*_(*s*, *s*′) is the probability that the sequence *s* evolved by descent with modification giving as a result the sequence *s*′.
2. Given *s*_1_, *s*_2_, *s*′ ∈ Σ*, *P*_*H*_(*s*_1_, *s*_2_, *s*′) is the probability that a hybridization between sequences *s*_1_ and *s*_2_ –possibly coupled with descent with modification– gives as result the sequence *s*′.

For each tree node *t* of *N*, we let *ϕ*_*t*_: Σ* → [0, 1] be the function defined as follows. If *t* is the root of *N*, then *ϕ*_*t*_ is the constant function equal to 1. Otherwise, if the single parent *p* of *t* is a tree node, then *ϕ*_*t*_(*s*) = *P*_*S*_(*s*(*p*), *s*). If *p* is a hybrid node with parents *g*_1_, *g*_2_, then *ϕ*_*t*_(*s*) = *P*_*H*_(*s*(*g*_1_), *s*(*g*_2_), *s*). That is, *ϕ*_*t*_(*s*) is the probability that a given sequence *s* is the result of the evolution of the sequences at the parent node (or grandparents, in case of hybrid parent) of *t*.

Now, we want to extend *N* to another BTC network in order to include an extant OTU *ℓ* ∉ *X* identified by its sequence *s*_*ℓ*_ ∈ Σ*, while keeping the sequences associated to all tree nodes of *N*. According to the results presented in this paper, we need to identify the augmentation operation *R*^−1^(*N*, *ℓ*; *S*_1_, *S*_2_) that has to be applied, and determine the sequences at the newly created tree nodes. If the operation to be applied is of type *T*, that is, $\tilde{N}=T^{-1}\left(N,\ell ;S_{1},S_{2}\right)$, we need to find certain nodes *τ*_1_, *y*_1_, …, *y*_*r*−1_, with the additional condition that *S*_1_ = {*τ*_1_} and *S*_2_ = (*y*_1_, …, *y*_*r*−1_) form a *T*-feasible pair. Analogously, if it is of type *H*, $\tilde{N}=H^{-1}\left(N,\ell ;S_{1},S_{2}\right)$, then *S*_1_ = {*τ*_1_, *τ*_2_} and *S*_2_ = (*y*_1_, …, *y*_*r*−1_) must form a *H*-feasible pair.

Intuitively, the node *τ*_1_ in case of an augmentation of type *T*, or the nodes *τ*_1_ and *τ*_2_ in case of type *H*, have to be chosen in order to maximize the probability of appearance of the new OTU, while the other nodes appear in order to give a better explanation of the corresponding sequences by means of hybridization with the lineage leading to *ℓ*.

**Fig 15:**
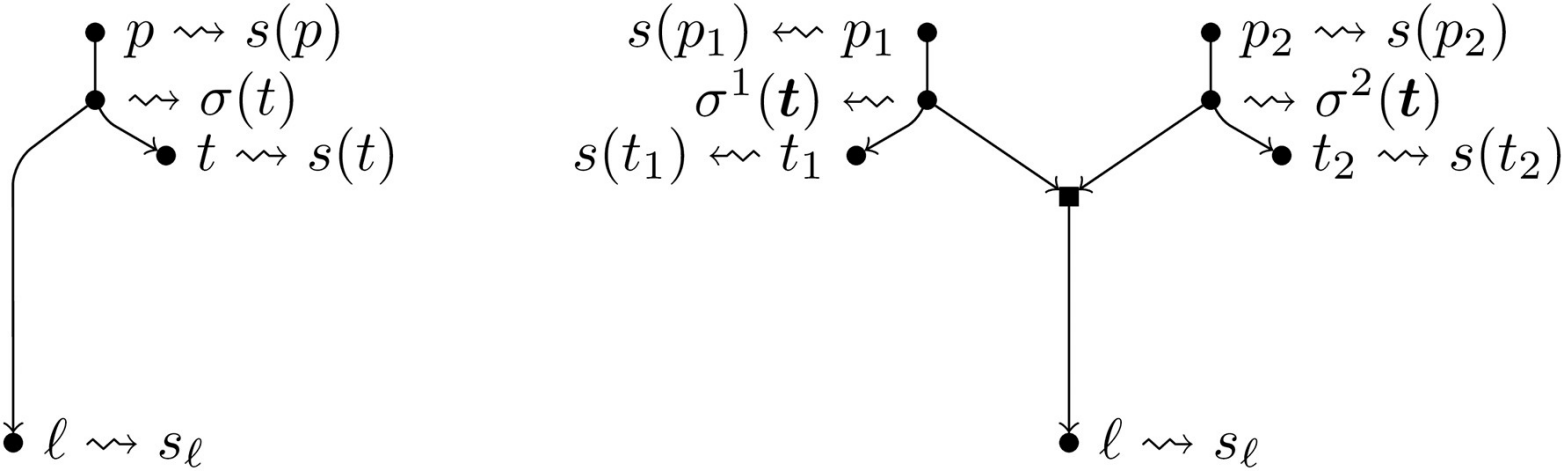
A depiction of the notations used in the text to define the function *π* to maximize for finding *τ*_1_ (left, type *T* augmentation) and {*τ*_1_, *τ*_2_} (right, type *H* augmentation). Although in the figure *p*, *p*_1_ and *p*_2_ are depicted as tree nodes, they can as well be hybrid nodes.

**Fig 16:**
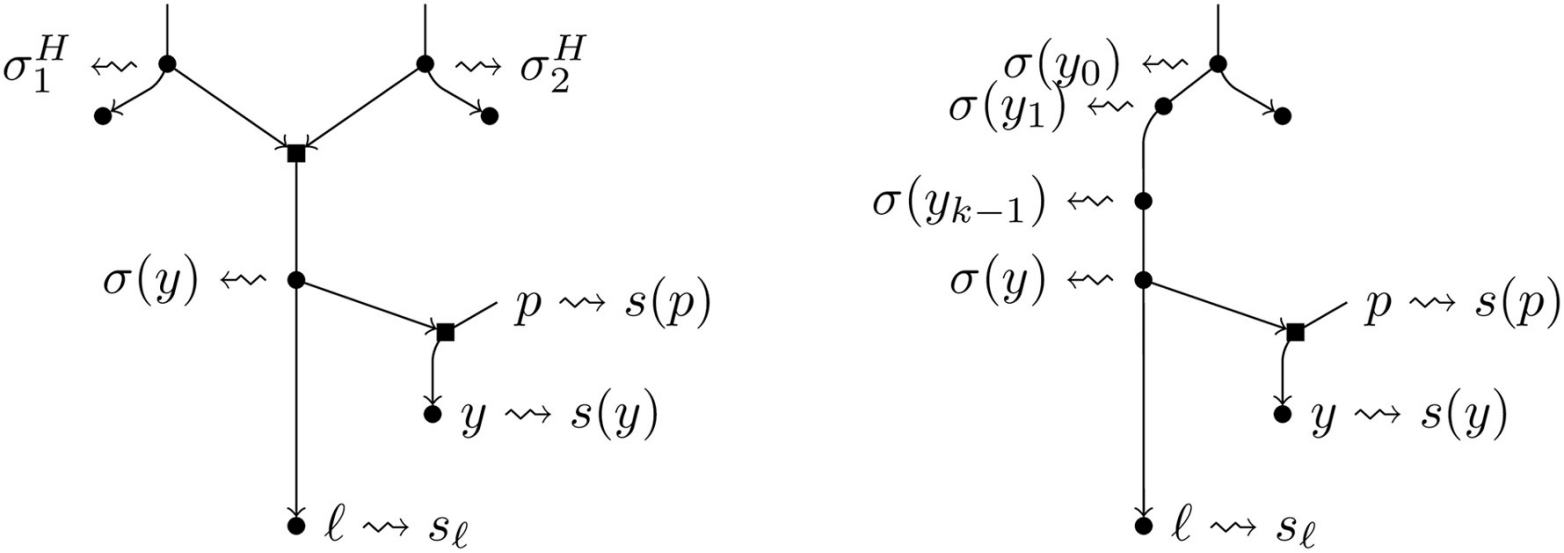
A depiction of the notations used in the text to define the function *π* to maximize for finding *y*_*k*_, respectively for a type *H* augmentation (left) and a type *T* augmentation (right), assuming that *y*_1_, …, *y*_*k*−1_ are already determined.

We present here an heuristic to find the augmentation operation, together with the assignment of sequences to new tree nodes, that deploys this intuitive idea:

1. Assume that an augmentation of type *T* is going to take place. To determine *τ*_1_, for each tree node *t* of *N*, we find a sequence *σ*(*t*) ∈ Σ* that maximizes
  $$\begin{array}{c} \pi \left(t\right)=\phi_{t}\left(\sigma \left(t\right)\right)\cdot P_{S}\left(\sigma \left(t\right),s\left(t\right)\right)\cdot P_{S}\left(\sigma \left(t\right),s_{\ell }\right). \end{array}$$
  The rationale behind this expression is that we look for the best way to divide the arc entering *t* to add the new taxon with sequence *s*_*ℓ*_ as child of the newly created node. See Fig 15(left) for a depiction of this. Then *τ*_1_ is a node with the maximum value of *π* over all nodes of *N*, that is the best location where to hang *s*_*ℓ*_ in *N*. For future reference, let *σ*^*T*^ = *σ*(*τ*_1_), *π*^*T*^ = *π*(*τ*_1_) and $S_{1}^{T}=\left\{\tau_{1}\right\}$.
2. Assume that an augmentation of type *H* is going to take place. To determine *τ*_1_, *τ*_2_, for each unordered pair of tree nodes {*t*_1_, *t*_2_} ≔ ***t*** of *N*, we find sequences *σ*^1^(***t***), *σ*^2^(***t***) ∈ Σ* that maximize
  $$\begin{array}{c} \pi \left(t\right)=\phi_{t_{1}}\left(\sigma^{1}\left(t\right)\right)\cdot P_{S}\left(\sigma^{1}\left(t\right),s\left(t_{1}\right)\right)\cdot \phi_{t_{2}}\left(\sigma^{2}\left(t\right)\right)\cdot P_{S}\left(\sigma^{2}\left(t\right),s\left(t_{2}\right)\right)\cdot P_{H}\left(\sigma^{1}\left(t\right),\sigma^{2}\left(t\right),s_{\ell }\right) \end{array}$$
  See Fig 15(right). The rationale for this choice is the same as in the previous point.
  Let ***τ*** = {*τ*_1_, *τ*_2_} be a pair with maximum value of *π*. For future reference, let $\sigma^{H}=\left(\sigma_{1}^{H},\sigma_{2}^{H}\right)=\left(\sigma^{1}\left(\tau \right),\sigma^{2}\left(\tau \right)\right)$, *π*^*H*^ = *π*(***τ***) and $S_{1}^{H}=\tau$.
3. If *π*^*T*^ ≥ *π*^*H*^, we opt for an augmentation of type *T* and we let $S_{1}=S_{1}^{T}$; otherwise, we opt for an augmentation of type *H* and we let $S_{1}=S_{1}^{H}$. The tree nodes in *R*^−1^(*N*, *ℓ*; *S*_1_, ∅) that were present in *N* keep their sequences. Moreover, in case of an augmentation of type *T*, we subdivide the arc entering *τ*_1_ via a new node *w*_1_ associated to the sequence *σ*^*T*^ and we add a new leaf *ℓ* with sequence *s*_*ℓ*_ as child of *w*_1_. In case of an augmentation of type *H*, we subdivide the arcs entering *τ*_1_ and *τ*_2_ via two new nodes *w*_1_, *w*_2_ that are assigned to the two sequences $\sigma_{1}^{H},\sigma_{2}^{H}$; then, we add a new hybrid node with parents *w*_1_ and *w*_2_ and having as child a new leaf *ℓ* with sequence *s*_*ℓ*_.
4. For each *k* ≥ 1, we assume that *y*_1_, …, *y*_*k*−1_ are already determined. Let *C* be the set of tree nodes *y* such that (*S*_1_, (*y*_1_, …, *y*_*k*−1_, *y*)) is a feasible pair. To determine *y*_*k*_ we proceed as follows:
  - If *k* = 1 and we opted for a type *H* augmentation, for each *y* ∈ *C* with parent *p*, we find a sequence *σ*(*y*) that maximizes
    $$\begin{array}{c} \pi \left(y\right)=P_{H}\left(\sigma_{1}^{H},\sigma_{2}^{H},\sigma \left(y\right)\right)\cdot P_{H}\left(\sigma \left(y\right),s\left(p\right),s\left(y\right)\right)\cdot P_{S}\left(\sigma \left(y\right),s_{\ell }\right). \end{array}$$
    See Fig 16(left). Notice that we have assumed that *p* is a tree node; if *p* was a hybrid node, and this case is really exceptional because of the definition of feasible pair, then the computation above, and the one in the next item, should be adapted. If *κ*(*y*) = *π*(*y*) − *P*_*S*_(*s*(*p*), *s*(*y*)) $P_{H}\left(\sigma_{1}^{H},\sigma_{2}^{H},s_{\ell }\right)$ is negative, we remove *y* from *C* since this means that the hypothesis of the existence of a hybridization just above *y* is less likely than its absence.
  - If *k* > 1 or we opted for a type *T* augmentation, for each *y* ∈ *C* with parent *p*, we find a sequence *σ*(*y*) that maximizes
    $$\begin{array}{c} \pi \left(y\right)=P_{S}\left(\sigma \left(y_{k-1}\right),\sigma \left(y\right)\right)\cdot P_{H}\left(\sigma \left(y\right),s\left(p\right),s\left(y\right)\right)\cdot P_{S}\left(\sigma \left(y\right),s_{\ell }\right), \end{array}$$
    where in case that *k* = 1 (and hence we opted for a type *T* augmentation), we let *y*_0_ = *τ*_1_. See Fig 16(right). If *κ*(*y*) = *π*(*y*) − *P*_*S*_(*s*(*p*), *s*(*y*))*P*_*S*_(*σ*(*y*_*k*−1_), *s*_*ℓ*_) is negative, we remove *y* from *C* as in the previous case.
  Then, if *C* is empty we output the network $\tilde{N}=R^{-1}\left(N,\ell ;S_{1},\left(y_{1},\ldots ,y_{k-1}\right)\right)$. Otherwise, let *y*_*k*_ the node that maximizes *κ*. Then, we create a new tree node *u*_*k*_ on the arc entering *ℓ* –associating the sequence *σ*(*y*_*k*_) to it– and we subdivide the arc entering *y*_*k*_ by a new hybrid node with second parent *u*_*k*_. The tree nodes in *R*^−1^(*N*, *ℓ*; *S*_1_, (*y*_1_, …, *y*_*k*−1_, *y*_*k*_)) that appeared in *R*^−1^(*N*, *ℓ*; *S*_1_, (*y*_1_, …, *y*_*k*−1_)) keep their associated sequence.

We emphasize that we do not claim that the heuristic we present here gives a global optimum. In fact, usually a sequence of optimal choices does not lead to a global optimum. The analysis, and eventually improvement, of this method of reconstruction is left as future work.

**Example 1**. We consider a simple model of evolution where:

- OTUs are represented by words of length 4 in the alphabet Σ = {A, B, C}.
- For speciation we assume a simple Jukes-Cantor model of evolution on the characters A, B, C so that *P*_*S*_(*s*, *s*′) = *μ*^*d*^(1 − 2*μ*)^4−*d*^, where *d* = *d*(*s*, *s*′) is the Hamming distance between *s* and *s*′ and *μ* < 1/3 is a parameter of the model.
- In this toy example, we model hybridizations as if they were plain recombinations where half of the hybrid sequence comes from one parent and the other half from the other. So, given two sequences $s^{1}=\left(s_{1}^{1},s_{2}^{1},s_{3}^{1},s_{4}^{1}\right)$ and $s^{2}=\left(s_{1}^{2},s_{2}^{2},s_{3}^{2},s_{4}^{2}\right)$, *P*_*H*_(*s*^1^, *s*^2^, *s*′) = 1/2 if $s^{\prime }=\left(s_{1}^{1},s_{2}^{1},s_{3}^{2},s_{4}^{2}\right)$ or $s^{\prime }=\left(s_{1}^{2},s_{2}^{2},s_{3}^{1},s_{4}^{1}\right)$, and *P*_*H*_(*s*^1^, *s*^2^, *s*′) = 0 otherwise.

We consider three species, with sequences

$$\begin{array}{c} s(\alpha )=\text{AAAC},\;s(\beta )=\text{BBCC},\;s(\gamma )=\text{BBBB}. \end{array}$$

**Fig 17:**
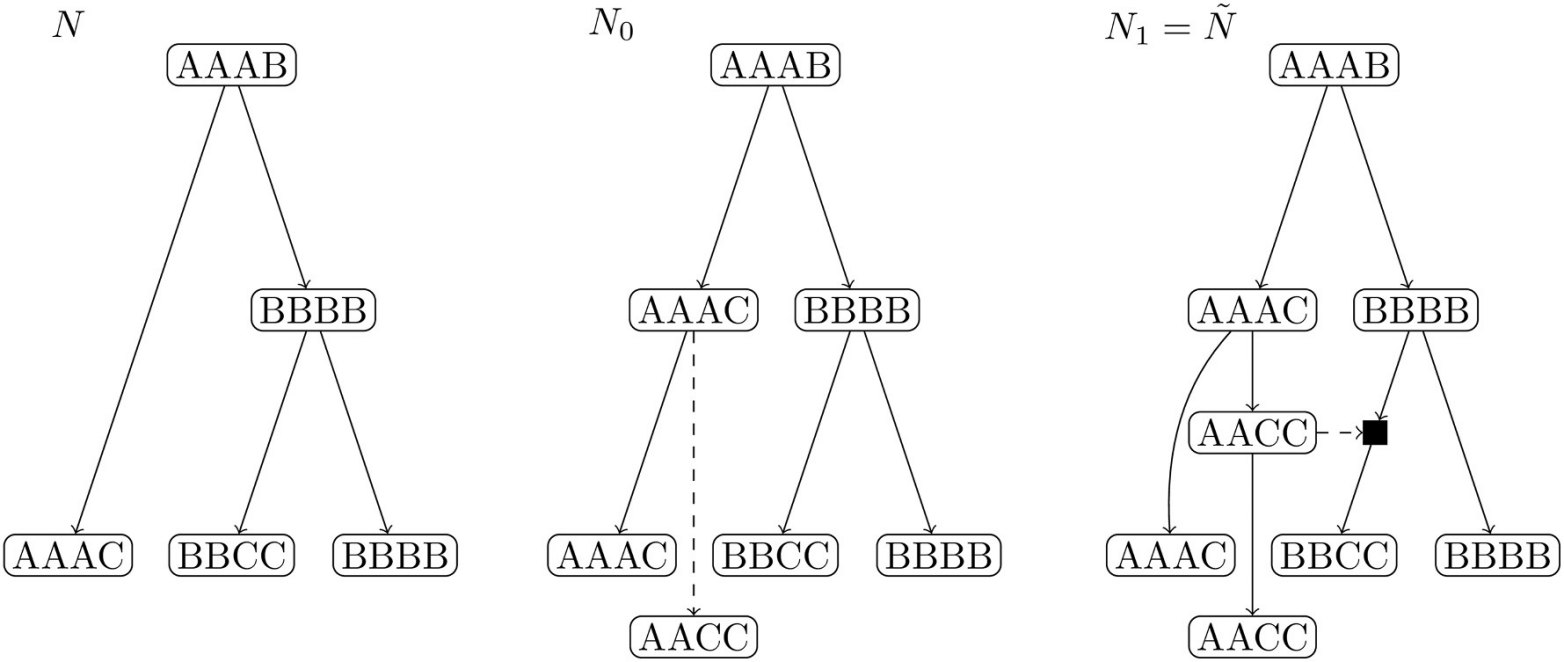
The networks considered in Example 1. We start with a tree on three leaves (left), chose a type *T* augmentation and find the best choice for *τ*_1_ (middle) and the best choice for the vector (*y*_1_, …, *y*_*k*_) (right, here *k* = 1).

The network *N* (which is in fact a tree) that fits these extant OTUs best, together with an optimal assignment of sequences to all nodes is shown in Fig 17(left).

Now, we wish to extend *N* in order to add a new OTU with sequence

$$\begin{array}{c} s(\delta )=\text{AACC}. \end{array}$$

We thus proceed as discussed in the previous pages:

1. If we assume an augmentation of type *T*, it is not difficult to see that an optimal choice for *τ*_1_ and its corresponding sequence *σ*^*T*^ are respectively *α* and AAAC, with *π*(*α*) = *μ*^2^(1 − 2*μ*)^10^.
2. If we explore the different possibilities for an augmentation of type *H*, we find that the best choice is {*τ*_1_, *τ*_2_} = {*α*, *β*} with sequences AAAB and BBCC and with value *π*({*τ*_1_, *τ*_2_}) = *μ*^3^(1 − 2*μ*)^13^/2.
3. Since *μ* < 1/3, we get that *π*(*α*) > *π*({*α*, *β*}) and we opt for the augmentation *N*_0_ = *T*^−1^(*N*, *δ*;{*α*}, ∅) shown in Fig 17(middle).
4. Now we want to find the right choice for *y*_1_, if any. All nodes except the least common ancestor (in *N*_0_) of *α* and *δ* are in *C*. Taking any node *y* ∈ *C* different from *β* we get that *κ*(*y*) < 0 and hence they are not good candidates. If we take *y* = *β*, taking into account that *μ* < 1/3, we find an optimal sequence *σ*(*β*) = AACC. The value of *π*(*β*) corresponds to three different evolution processes: two mutations, from AAAC to AACC and from AACC to AACC, and a hybridization of the sequences AACC and BBBB to BBCC, and hence we get *π*(*β*) = (1 − 2*μ*)^7^ *μ*/2. Now, this value must be compared with the probability of evolution without this hybridization, i.e. the probability of speciations from BBBB to BBCC and from AAAC to AACC which is *μ*^3^(1 − 2*μ*)^5^. Since (1 − 2*μ*)^7^ *μ*/2 > *μ*^3^(1 − 2*μ*)^5^ (assuming that *μ* < 0.2928, which is a reasonable assumption) we conclude that the network *N*_1_ = *T*^−1^(*N*, *δ*; {*α*}, (*β*)), depicted in Fig 17(right), is a better explanation than *N*_0_. Hence, we let *y*_1_ = *β*.
5. If we repeat the procedure in the previous step, we find that no hybridization improves the probability of the sequences, giving as final result the network $\tilde{N}=T^{-1}\left(N,\delta ;\left\{\alpha \right\},\left(\beta \right)\right)$ in Fig 17(right).

### Computational experiments

The algorithms in this paper have been implemented in python using the python library PhyloNetworks [28]. This implementation, together with the sources for the experiments that we comment in this subsection can be downloaded from https://github.com/bielcardona/TCGenerators.

#### Exhaustive and sequential construction of networks in ${BTC}_{n}$

We have implemented both the exhaustive and sequential construction of BTC networks with *n* leaves. The number of such networks increases very rapidly, and hence the exhaustive construction is not feasible for *n* ≥ 8. For *n* ≤ 7 we generated all the networks in ${BTC}_{n}$; see Table 1 for the number of such networks. For *n* = 8 we could not compute them all, since there are around twelve trillion of such networks: We took uniform samples of networks in ${BTC}_{7}$ and computed their respective offspring, and repeated this procedure until the average number of offsprings per network stabilized up to 4 digits; this allowed us to give the estimate for $|{BTC}_{8}|$.

**Table 1:** Exact number of BTC networks over [*n*] for *n* = 1, …, 7, an estimate for *n* = 8, and their upper bounds for *n* ≤ 10.

*n*	$|{BTC}_{n}|$	upper bound
1	1	1
2	3	3
3	66	85
4	4,059	7,442
5	496,710	1,317,098
6	101,833,875	387,405,870
7	31,538,916,360	169,781,857,790
8	≃ 12,000,000,000,000	103,409,407,515,286
9	?	83,400,205,845,281,275
10	?	85,947,517,732,640,544,027

#### Random construction of networks in ${BTC}_{n}$

We have implemented the following construction, that does not generate networks uniformly, but is the closest we could get to it. We start with the network *N*_1_ with a single node labeled by 1. At each stage *i* = 1, …, *n* − 1, we explicitly find all feasible pairs inside *N*_*i*_ and choose at random and uniformly one of them to generate the network *N*_*i*+1_. This procedure generates all possible networks in ${BTC}_{n}$, but not uniformly, since different networks over the same set of taxa may have different numbers of feasible pairs.

#### Computation of bounds for $|{BTC}_{n}|$

Finally, we have implemented the recursive computation for the upper bounds of $|{BTC}_{n}|$ using the bounds for the offsprings of BTC networks found previously. The results for *n* up to 10 are given in Table 1, where it is observed that, at least for small values of *n*, the true number of networks and the upper bounds have the same order of magnitude.

## Discussion

The main result of this paper is a systematic way of recursively generating, with unicity, all BTC networks with a given number of leaves. This procedure relies on a pair of reduction/augmentation operations that generalize analogous operations for phylogenetic trees. Indeed, given a (rooted, binary) phylogenetic tree over [*n*], we can obtain a phylogenetic tree over [*n* − 1] by deleting the leaf labeled by *n* and removing the elementary node that this deletion generates. Conversely, given a tree *T* over [*n* − 1] and one of its nodes *u*, we can construct a tree over [*n*] by simply hanging a pendant leaf labeled by *n* to the single incoming arc of *u*. Since different choices for *T* and *u* give different trees over [*n*], this gives a recursive procedure to generate, with unicity, all binary rooted phylogenetic trees over a given set of taxa: we start with the leaf labeled by 1, then we add the leaf labeled by 2, then the leaf labeled by 3 in all possible ways, and so on. Biologically, we can think of this procedure as follows: Once the evolutionary history of a given set of OTUs is correctly established (notice that, in practice, we can never be sure that we got the correct tree, but here we suppose we do) and modeled by a phylogenetic tree, extending this evolutionary history to consider a “new” OTU *n* consists in finding where to place *n* in the tree, i.e. finding the speciation event that leads to the diversification of *n*.

Unfortunately, when working with classes of phylogenetic networks, the removal of a single leaf (and of all elementary nodes created by this removal) does not necessarily give a phylogenetic network within the same class. In the case of BTC networks, we were able to find the minimal set of nodes that one must remove so that, after their deletion and that of all elementary nodes created by this removal, one gets a BTC network with one leaf less. As in the case of trees, given a BTC network over [*n* − 1] and some set of nodes with certain restrictions (i.e. the feasible pairs *S*_1_ and *S*_2_) we can construct a BTC network over [*n*] leaves, in such a way that different choices for the BTC network or for the feasible pair give different BTC networks over [*n*]. Hence, we find a procedure to recursively generate all BTC networks over a given set of taxa. Biologically, we can think of this procedure as an extension of what can happen when adding a new OTU *n* to a phylogenetic tree: here the diversification of *n* can involve a reticulated event (when *n* is added as hybrid node) and the ancestors of *n* participate to |*S*_2_| reticulated events, which were impossible to detect before the introduction of *n*.
