# Correction: Generation of Binary Tree-Child phylogenetic networks

**DOI:** 10.1371/journal.pcbi.1007440

**Published:** 2019-10-09

**Authors:** Gabriel Cardona, Joan Carles Pons, Celine Scornavacca

There is a funder missing from the Funding statement. The full statement should read: “Research of GC and JCP has been partially supported by the Spanish Ministry of Science, Innovation and Universities (http://www.ciencia.gob.es/), Spanish State Research Agency (http://www.ciencia.gob.es/portal/site/MICINN/aei) and European Regional Development Fund (https://ec.europa.eu/regional_policy/es/funding/erdf/) projects DPI2015-67082-P and PGC2018-096956-B-C43. The funders had no role in study design, data collection and analysis, decision to publish, or preparation of the manuscript.”

## References

[1] Cardona G, Pons JC, Scornavacca C (2019) Generation of Binary Tree-Child phylogenetic networks. PLoS Comput Biol 15(9): e1007347 10.1371/journal.pcbi.1007347 31509525 PMC6756559

